# Physiological Changes, Activity, and Stress During a 100-km–24-h Walking-March

**DOI:** 10.3389/fphys.2021.640710

**Published:** 2021-03-11

**Authors:** Marc Jörres, Hanns-Christian Gunga, Mathias Steinach

**Affiliations:** Charité – Universitätsmedizin Berlin, corporate member of Freie Universität Berlin, Humboldt-Universität zu Berlin, and Berlin Institute of Health, Institute of Physiology, Center for Space Medicine and Extreme Environments, Berlin, Germany

**Keywords:** body composition, cardiac stress, endurance, exercise performance, low intensity, physiological changes, ultra-endurance, ultramarathon

## Abstract

**Background:**

Long-endurance exercises like ultramarathons are known to elicit various metabolic and physiological changes in the human body. However, little is known about very long-duration exercise at low intensities regarding healthy human subjects.

**Aim:**

The purpose of this study was to evaluate changes in body composition and metabolism in long-endurance but low-intensity events.

**Methods:**

Twenty-five male and 18 female healthy recreational athletes (age 34.6 ± 8.8 years; BMI: 22.4 ± 2.0 kg/m^2^) of the “100 km Mammutmarsch” were recruited for participation during the events in 2014–2016. Other than classical ultramarathons, the “Mammutmarsch” is a hiking event, in which participants were required to walk but not run or jog. It was expected to complete the 100-km distance within 24 h, resulting in a calculated mean speed of 4.17 km/h, which fits to the mean speed observed (4.12 ± 0.76 km/h). As not all participants reached the finish line, comparison of finishers (FIN, *n* = 11) and non-finishers (NON, *n* = 21) allowed differential assessment of performance. Body composition measured through bioelectrical impedance analysis (BIA) was determined pre- and post-event, and serum samples were taken pre-event, at 30, 70, and 100 km to determine NT-pro-BNP, troponin T, C-reactive protein (CRP), cortisol, low-density lipoprotein (LDL), high-density lipoprotein (HDL), triglycerides, total cholesterol, total creatine kinase (CK), CK-MB, aminotransferase (AST), ALT, and sodium levels. Nineteen participants wore actimeter armbands (SenseWear^®^) to gain information about body activity and exercise intensity [metabolic equivalent of task (MET)]. Sixteen participants wore mobile heart rate monitors to assess mean heart rate during the race. Serum parameter alterations over the course of the race were analyzed with mixed-effects ANOVA and additional *t*-tests. All serum parameters were analyzed for correlation concerning different MET levels, speed, age, BMI, baseline NT-pro-BNP, mean heart rate during the race, and sex with linear regression analysis.

**Results:**

We found significant elevations for muscle and cardiac stress markers (CRP, CK, CK-MB, AST, ALT, cortisol, and NT-pro-BNP) as well as decreasing markers of lipid metabolism (cholesterol, triglycerides, LDL). Although the intensity level demanded from our participants was low compared with other studies on (ultra-) marathons, the alteration of tested parameters was similar to those of high-intensity exercise, e.g., NT-pro-BNP showed a fourfold increase (*p* < 0.01) and LDL decreased by 20% (*p* = 0.05). Besides the duration of exercise, age, BMI, training status, and sex are relevant parameters that influence the elevation of stress factors. Notably, our data indicate that NT-pro-BNP might be a marker for cardiovascular fitness also in healthy adults.

**Conclusion:**

This low-intensity long-endurance walk evoked a strong systemic reaction and large cell stress and shifted to a favorable lipid profile, comparable to higher intensity events. Despite increasing cardiac stress parameters, there were no indications of cardiac cell damage. Remarkably, the duration seems to have a greater influence on stress markers and metabolism than intensity.

## Introduction

The knowledge about physiological changes in athletes participating in ultramarathons is scarce. While thousands of athletes compete in classic marathons, only a few hundred do so in ultramarathons, i.e., distances longer than 42.195 km. Nevertheless, in the last decades, the number of such events has been increasing, as well as the number of athletes participating in such events (Whyte, 2014; International Association of Ultrarunners, 2020; Knechtle et al., 2020).

Several studies have shown that engaging in endurance sports elicits various metabolic and physiological changes in the human body. Changes in lean body mass and body fat (Knechtle et al., 2008a; Baur et al., 2016), as well as a decrease in serum lipids (Ginsburg et al., 1996), are considered beneficial on diseases caused by western sedentary lifestyle, such as the metabolic syndrome (Saklayen, 2018), while other alterations, such as increases in cardiac and muscle cell damage markers and markers indicating systemic inflammatory reaction, may indicate adverse effects (Fallon, 2001; Scharhag et al., 2005, 2006; Kasprowicz et al., 2013; Kłapcińska et al., 2013; Baur et al., 2016). Established markers to assess these changes are creatine kinase (CK), transaminases, C-reactive protein (CRP), and cortisol (Allen et al., 2015; Cerqueira et al., 2020). Markers to assess cardiac stress are NT-pro-BNP, released by cardiomyocytes in response to stretching caused by volume load (Nakagawa et al., 1995), and cardiac troponins, indicating myocardial damage (Sarko and Pollack, 2002). These changes are discussed to be a physiological response as cardiac and muscle cell markers return to baseline shortly after finishing a marathon or ultramarathon, suggesting rapid cell repair without adverse long-term effects (Scharhag et al., 2005, 2006, 2008; La Gerche et al., 2008; Kłapcińska et al., 2013).

However, the majority of these studies assessed triathlons, marathons, or ultramarathons where participants ran or jogged (Ohba et al., 2001; Wu et al., 2004; Scharhag et al., 2005; Scott et al., 2009; Banfi et al., 2012; Kim et al., 2014; Salvagno et al., 2014; Vilela et al., 2015; Emed et al., 2016; Yoon et al., 2016). In those races, exercise intensity and, therefore, mean speed is usually relatively high, e.g., around 14 km/h in 80-km races (Zingg et al., 2015). In studies with lower mean running speed around 5 km/h, distances were much longer (up to 308 km), but participants still ran or jogged instead of walking (Kim et al., 2014; Yoon et al., 2016). One study with a relatively low mean speed covered a distance of up to 430 miles (≈690 km) but was conducted in extremely cold weather (daily average temperatures around −25°C) (Coker et al., 2017).

Research concerning NT-pro-BNP in endurance exercise showed levels exceeding its upper reference limit (URL) in 35.9% of runners; however, only marathon and half-marathon races were assessed (Vilela et al., 2015). Very few studies exist that evaluated changes in NT-pro-BNP during ultramarathons. The distances in existing studies varied between 60 (Salvagno et al., 2014), 100 (Scharhag et al., 2005), 160 (Scott et al., 2009), and 280 km (Kłapcińska et al., 2013) up to 308 km (Kim et al., 2014; Yoon et al., 2016) with running velocities varying from 8.5 to 10.0 km/h (Kasprowicz et al., 2013), respectively. Two studies (Kim et al., 2014; Yoon et al., 2016) evaluating NT-pro-BNP at lower intensities (i.e., walking instead of jogging or running) covered more than triple the distance than the MM, assessed in the present study. Another study covering exercise at low intensities did not include healthy subjects but examined patients with coronary heart syndrome, evaluated for only 30 min (Scharhag et al., 2007). To our knowledge, no data covering healthy athletes walking for a distance around 100 km exists.

Cardiac troponins have been analyzed in many studies regarding half-marathon, marathon, and ultramarathon-runs with increases in 51% of 1,045 evaluated athletes above the cut-off value of 10 ng/L (Sedaghat-Hamedani et al., 2015). However, participants of the evaluated ultramarathons were running at jogging speed (9–10 km/h at a distance of 100 km) (Ohba et al., 2001; Scharhag et al., 2005). Only few studies have evaluated troponins at low-intensity and long-duration exercise. However, in these few studies, participants were either heart patients (Benda et al., 2016); were 55 years in mean age of which 82% were prescribed diuretics, statins, or antihypertensive drugs; or were of heterogenic age (21–82 years) of which 23% had cardiac pathologies (Eijsvogels et al., 2010).

Regarding metabolic and stress parameters, several studies have shown that ultramarathons may lead to a catabolic state and fatigue with a decrease in triglycerides and cholesterol (Emed et al., 2016) and increases of up to fourfold in cortisol (Baur et al., 2016), 100,000 IU/L in CK, 40-fold in CRP, and 20-fold in aspartate aminotransferase (AST) (Wu et al., 2004; Baur et al., 2016). At present, only one study had included low-intensity long-duration exercise at 60% of the participants’ VO_2_max, but included different disciplines like walking, cycling, and kayaking (Marklund et al., 2013).

Thus, previous studies assessed multiple changes during ultramarathons in which athletes tried to reach their personal limits, e.g., running or jogging the longest possible distance in 48 h (Kłapcińska et al., 2013) or finish a certain distance as fast as possible (Scharhag et al., 2005; Scott et al., 2009; Kim et al., 2014; Salvagno et al., 2014; Baur et al., 2016; Yoon et al., 2016). As walking and running speed correlate with exercise intensity [e.g., expressed in metabolic equivalent of task (MET)] (Ainsworth et al., 1993; Mendes et al., 2018), the literature lacks information concerning long-endurance exercise of low intensity, i.e., walking instead of running among healthy human adults. While high-intensity endurance exercises are clearly associated with the elevation of diverse stress markers, it is questionable whether low-intensity exercise over long distances might be less stressful and, thus, more suitable for persons with pre-existing conditions, or if such events of low intensity elicit significant changes among metabolic and stress parameters in healthy humans at all.

In addition, the number of persons living a sedentary lifestyle is increasing (Arocha Rodulfo, 2019), leading to numerous adverse health effects like obesity and metabolic disorders (Mayer-Davis and Costacou, 2001) where this western sedentary lifestyle could be construed as an adverse environmental factor (Wachira et al., 2018; Koohsari et al., 2020; Lin et al., 2020).

The purpose of this study was, therefore, to evaluate if and to what extent changes in body composition and a wide range of serum markers are evoked in healthy adult participants by a 24-h–100-km walking–march of low intensity. In addition, we aimed to assess whether there were any correlations to individual intensity, age, sex, and BMI.

It was the null hypothesis of this study that there would be no significant changes in the evaluated parameters evoked by the low-intensity exercise and that individual intensity, age, sex, and BMI would not significantly influence the evaluated parameters. The findings might further contribute to our understanding of physiological processes of healthy human adults in response to low-intensity long-duration exercise and could help recreational athletes in their training as well as being applied in clinical settings with respect to diseases of western sedentary lifestyle.

## Materials and Methods

### Event

The “Mammutmarsch” (German for “Mammoth March,” henceforth “MM”) is an ultramarathon challenging participants to complete 100 km in less than 24 h (Mammutmarsch, 2018; Maggioni et al., 2020). Other than in classical (ultra-) marathons, the goal is not to finish the event as fast or as far as possible to cure a winner, instead the participants are required to only walk the entire distance. Therefore, the MM addresses leisure athletes rather than professional (ultra-) marathon runners. As the event is designed as a “march” without running or jogging, it is leading to a continuous long-duration exercise of low intensity, which marks the unique setting of this event. In 2016, around 2,000 athletes participated. The track consists of paved roads, gravel roads, soft forestry paths, or cobblestones leading through partly flat and partly hilly terrain into the rural areas around the city of Berlin. As shown by GPS data from 2015 to 2016, the track has an overall ascend and descend from 600 to 700 m each, with an altitude profile from 7 to 85 m. In 2014, the march led through the same terrain, with the same endpoint (Gusow, Brandenburg, Germany).

Regardless of checkpoints along the track providing snacks and water as well as benches to rest, all participants were responsible for their own gear and provisions and thus carried around 2–4 kg of equipment in a backpack.

Handicaps of the march were the long distance, the lack of sleep, and cool temperatures especially at night. The events that were part of this study took place on May 31/June 01, 2014, May 9/10, 2015, and May 14/15, 2016, starting at 5 pm and finishing 5 pm the next day. Temperatures ranged from 6°C at night to 19°C during the day with average temperatures of 14.3°C in 2014, 13.3°C in 2015, and 9.3°C in 2016. In 2014 and 2015, conditions were dry for 24 h, while in 2016, there was light drizzle (2 mm over 3 h) at the end of the race.

As the conditions for all 3 years were similar concerning temperature, weather, track, and season, all participants’ data were pooled for statistical analysis.

### Subjects

This study includes 43 healthy individuals of which were 25 male and 18 female. All participants attended in the MM 2014, 2015, and 2016. Due to issues relating to serum sample data collection, out of the 43 subjects, 32 (19 male, 13 female) were included in the serum sample data analysis.

Recruitment was conducted with support of the event organizers. All participants received an email announcing the study with a call for participation. Interested individuals contacted the investigators *via* email and were provided with information regarding the purpose of the study, methods, and used equipment before enrolling. Participants were chosen in the order they applied and with the purpose to achieve an even distribution of females and males. There were no special requirements to partake in the study – all adult participants were eligible for inclusion in the study. All were recreational athletes; some had a background of taking part in similar marches or other long-endurance events.

The potential study participants were given appropriate time to consider partaking in the study and to raise questions or concerns. All gave their informed written consent to partake in the study.

The study was approved by the Charité Ethics Board (review number EA1/163/14), and all measurements and procedures complied with the Declaration of Helsinki (54th Revision 2008, Korea) regarding the treatment of human subjects.

### Measurements

#### Baseline Assessment

Body mass, height, BMI, body composition, resting heart rate, and serum parameters were measured pre-race in a standardized laboratory environment indoors in an air-conditioned laboratory (temperatures 18–21°C) the week prior to the event. To assess training status with regard to our study, the participants filled out a questionnaire about the distances covered per week on foot (including both walking and jogging) of the previous half year prior to the event and self-evaluated their endurance capability on a rating scale from 1 to 10. Subjects were advised to have fasted for 6–8 h and to abstain from sleep deprivation the previous night as well as alcohol and drug consumption (except for oral contraceptives for the female subjects) and from caffeine consumption the morning of the test. Directly before the measurements, participants were instructed to empty the bladder and to remove all jewelry for pre-race measurements.

Anthropometric data were gathered with a calibrated scale and height meter (Seca, Germany) on an even surface while participants wore only minimal clothing (light underwear). Mean resting heart rate was measured for all the participants with a mobile heart rate monitor [RS800CX, Polar (Williams et al., 2017)] in a supine position for 10 min.

Body composition was evaluated using bioelectrical impedance analysis (BIA) via the tetra polar electrode method with a BIA 101 device from AKERN, Italy (Lukaski and Bolonchuk, 1988; Jaffrin and Morel, 2008). The BIA 101 measures resistance at a fixed constant sine current of 50 kHz for the determination of reactance in human tissue and has been clinically validated to allow the evaluation of body composition (Segal et al., 1988) and provided a mobile platform of data collection during our study. For quantification of fat mass, lean tissue mass, and percent body fat, the formula of Segal et al. for non-obese subjects was used. The equation is validated to assess body composition in heterogeneous populations (Segal et al., 1988). To assess total body water, the formula of Sun et al. (2003) was applied. Measurements were conducted while the participants were in a supine position after having laid down for 10 min to allow equal redistribution of body fluids in all instances, with minimal clothing and removed metals. For all measurements, the right limbs were used. Undoubtedly incorrect calculations (one participant) were excluded.

Serum samples were taken from cubital vein blood collections in a Sarstedt S-Monovette^®^, immediately chilled on ice (without direct contact to the ice to avoid freezing), and stored in a sealed cooling box. Directly after the race, samples were centrifuged for 10 min at 2,000 × *g*. Afterward, they were stored overnight at 4°C and sent to the laboratory the next day. The parameters NT-pro-BNP (pg/ml), troponin T (ng/L), CRP (mg/L), cortisol (μg/dl), low-density lipoprotein (LDL) (mmol/L), high-density lipoprotein (HDL) (mmol/L), triglycerides (mmol/L), total cholesterol (mmol/L), total CK (U/L), CK-MB (U/L), AST (U/L), ALT (U/L), and sodium (mmol/L) were determined by a certified laboratory [Labor 28 GmbH, Berlin, Germany, accredited at the “DAkkS” (Deutsche Akkreditierungsstelle GmbH), the national accreditation body for the Federal Republic of Germany, pursuant to regulation (EC) number 765/2008 and the accreditation body act of Germany] (Deutsche Akkreditierungsstelle GmbH, 2020).

#### In-Race Measurements

Actimeter armbands (SenseWear^®^) were given to 20 participants in order to measure body activity parameters such as exercise intensity {metabolic equivalent of task [1 MET = energy expenditure with an oxygen consumption of 3.5 ml O_2_/kg/min, resembling resting metabolic rate equal to 1 kcal/kg/h (Ainsworth et al., 1993)]}, energy expenditure, steps, time of activity, and duration of wearing the armband. Especially in cooler environments (Van Hoye et al., 2014) and in light exercise (Santos-Lozano et al., 2017) – like in the presented study – the SenseWear armband can be considered an accurate tool for assessment of energy expenditure. Each participant was instructed how to wear the actimeter and to set a time stamp right before starting and finishing the march. The collected data were analyzed using the manufacturer’s software (SenseWear Professional 7.0). From these data, mean walking speed was determined.

To measure heart rate during the race, mobile heart rate monitors [RS800CX, Polar (Williams et al., 2017)] were handed out to 16 participants.

Serum samples were taken at 30 and 70 km checkpoints and stored as mentioned above, and the same parameters were determined as in the pre-race measurements. For the collection, a pavilion with a bench and a table were set up at the checkpoints. To reduce influence on serum parameters by consumed food or fluids, the participants were instructed to neither eat nor drink 1 h before arriving at the checkpoints.

#### Post-race Measurements

Only for participants who reached the 70- or 100-km checkpoint, post-measurements were performed.

Post-race measurements for participants who reached the 100-km checkpoint were carried out within 30 min at the finishing area; as for the earlier checkpoints, no foods or drinks were to be consumed 1 h before reaching the finish and until completing all measurements. For participants who dropped out at the 70-km checkpoint, all post-measurements were carried out there. The BIA measurements were conducted in the same manner as during baseline assessment. After all participants finished the march (or dropped out at an earlier stage), blood samples were transported and centrifuged for 10 min at 2,000 × *g*. They were stored overnight at 4°C and sent to the laboratory the next day.

### Statistical Analysis

Acquired data were analyzed using appropriate statistical tests (mixed-effects analysis, *t*-test, and linear regression for correlation analysis) with the statistical software GraphPad Prism^®^, Version 8, GraphPad Software Inc., United States.

We analyzed two groups accordingly to their performance: participants who were able to finish the entire MM (FIN) and participants who dropped out at an earlier stage (NON).

Differences in anthropometric data in FIN and NON-and in between sexes were analyzed with an unpaired *t*-test. Body composition pre- and post-race was analyzed with paired *t*-test.

All statistical analyses for serum parameters were carried out with mixed-effects ANOVA. This mixed model uses a compound symmetry covariance matrix and is fit using restricted maximum likelihood (REML). In the absence of missing values, this method gives the same *p* values and multiple comparisons tests as repeated measures ANOVA. In the presence of missing values (missing completely at random), the results can be interpreted like repeated measures ANOVA.

Additionally, to assess differences in between single time points, paired *t*-tests were performed in FIN and NON.

Overall, these were the following numbers of cases in each group:

– FIN: pre: *n* = 11; 30 km: *n* = 9; 70 km: *n* = 10; 100 km: *n* = 11

In 2014, only pre- and post-values were assessed, and in 2015, one finishing participant missed the tent for collection of blood samples at 30 km.

– NON: pre: *n* = 21; 30 km: *n* = 21; 70 km: *n* = 8; 100 km: *n* = 0

Means at different time points between groups (FIN; NON) were analyzed using unpaired *t*-tests.

All serum parameters were analyzed for correlation concerning different MET levels, speed, age, BMI, baseline NT-pro-BNP, mean heart rate during the race, and sex with linear regression analysis. Regression lines were analyzed regarding goodness of fit (*r*^2^), differences in increase or decrease of parameters between the evaluated groups (slope), and differences in overall levels of parameters between the evaluated groups (*y*-intercept = elevation). If slopes differed too much, it was not possible to calculate whether *y*-intercepts differed significantly (“not computable” = “n.c.”).

Data of participants reaching the finish line after the 24-h time limit were used for statistical analyses as well (*n* = 2).

Results are presented as means ± SD. Statistical significance was assumed at *p* < 0.05.

## Results

### Demographics and Performance

A total of 32 athletes out of the 43 (25 male, 18 female; age 33.4 ± 8.9 years; BMI: 23.6 ± 2.7 kg/m^2^; resting heart rate 66.7 ± 11.3 bpm) partaking in all three MM competitions delivered usable serum samples and were included in the statistical analysis. Out of those 32 athletes, 11 athletes were able to complete the 100-km course (FIN) and 21 competitors withdrew during the race (NON), due to fatigue or musculoskeletal complaints. Table 1 depicts demographic and anthropometric data, as well as resting heart rate and the history of weekly covered distance on foot of the half year prior to the MM and the self-rated endurance capability of FIN and NON as well as female and male participants.

**TABLE 1:** Anthropometric data and demographics.

	**FIN**		
	***m***	***f***	***p* (m vs. f)**
	
*n*	7	4	
Age (years)	35.910.8	33.84.8	0.72
Height (cm)	182.711.0	176.84.5	0.33
Weight (kg)	76.18.6	68.346.1	0.14
BMI (kg/m^2^)	22.81.1	21.10.5	< 0.02*
Resting heart rate (bpm)	69.05.3	56.7510.1	0.09
Distance covered per week (km)	39.55.1	79.327.2	0.11
Endurance capability (self-rating, 1–10)	7.70.2	7.50.5	0.73

	**NON**		
	***m***	***f***	***p* (m vs. f)**

*n*	12	9	
Age (years)	39.32.3	27.81.9	< 0.01*
Height (cm)	179.53.7	171.82.3	< 0.03*
Weight (kg)	82.52.7	64.42.9	< 0.01*
BMI (kg/m^2^)	25.50.5	21.80.7	< 0.01*
Resting heart rate (bpm)	63.17.2	66.711.3	0.41
Distance covered per week (km)	34.09.0	38.711.7	0.77
Endurance capability (self-rating, 1–10)	8.00.8	6.41.3	0.02*

	**FIN vs. NON**		
	**FIN**	**NON**	***p* (FIN vs. NON)**

n	11	21	
Age (years)	35.79,7	34.39.0	0.72
Height (cm)	180.59.4	176.28.2	0.18
Weight (kg)	72.368.5	74.812.7	0.58
BMI (kg/m^2^)	22.21.3	23.92.6	< 0.05^§^
Resting heart rate (bpm)	64.59.2	64.69.1	0.97
Distance covered per week (km)	55.438.6	36.629.7	0.18
Endurance capability (self-rating, 1–10)	7.60.2	7.10.3	0.32

*Depicted are anthropometric data and subject demographics for male (m) and female (f) subgroups in FIN and NON as well as a comparison between all subjects of FIN and NON (presented are means ± SD). ^∗^Significant difference between male and female. ^§^Significant difference between FIN and NON.*

In both groups, FIN and NON, males had a significantly greater BMI than females. Additionally, in NON, male subjects were significantly older, taller, and heavier than female subjects, and the FIN group showed a more homogenous distribution in demographic and baseline parameters. Individuals who were able to finish the race (FIN) had a significantly lower BMI than participants who dropped out at an earlier stage (NON).

On average, finishers needed 22.83 h to complete the race, while the fastest male finisher ended after 21.68 h and the slowest after 24.78 h. The fastest female completed the race in 21.75 h and the slowest in 23.32 h. Mean speed was 4.12 ± 0.76 km/h in both sexes.

Subjects of FIN (4.39 ± 0.26 km/h) were faster compared with subjects of NON (3.87 ± 0.98 km/h) as a trend (*p* = 0.19). The subjects with the highest and lowest mean speed were both subjects of NON, with a mean speed of 4.94 and 2.32 km/h, respectively. FIN had a greater history of endurance training per week as a trend (55.4 ± 38.6 vs. 36.6 ± 29.7 km; *p* = 0.18). Overall, participants self-rated their endurance capability (mean) with a 7.3 out of 10 points; there were no significant differences between FIN and NON (7.6 ± 0.2 vs. 7.1 ± 0.3, *p* = 0.32), but male subjects in NON-self-rated their endurance capability significantly higher than female subjects (8.0 ± 0.8 vs. 6.4 ± 1.3, *p* = 0.02).

### Body Composition

Participants reaching the 70-km checkpoint or the 100-km finish were included in this analysis (*n* = 18, 9 female, 9 male), and one participant who delivered faulty data was ruled out.

Pre-event mean body weight was 70.4 ± 10.3 kg with a mean BMI of 22.4 ± 2.0 kg/m^2^. Both weight and BMI decreased significantly post-event to 69.7 ± 10.2 kg (mean Δ = 0.7 kg; *p* < 0.01) and 22.1 ± 2.0 kg/m^2^ (*p* < 0.01), respectively. BIA testing revealed significant changes: lean body mass decreased from 57.1 ± 10.5 to 56.2 ± 10.3 kg (*p* < 0.01) that correlated with a loss in total body water (42.1 ± 8.5 L before vs. 41.3 ± 8.1 L after MM; *p* < 0.01) (Mayer-Davis and Costacou, 2001). According to the fat-specific formula (male < 20%; female < 30% body fat) of Segal et al., this represents a loss of approximately 1.6% (0.9 kg) and 1.9% (0.8 L), respectively. The percentage of body fat did not change significantly (Table 2).

**TABLE 2:** Body composition pre- and post-race.

	**Body composition**		
	**Pre**	**Post**	***p* (pre vs. post)**
*n*	18	18	
Body mass (kg)	70.410.3	69.710.2	< 0.01*
BMI (kg/m^2^)	22.42.0	22.12.0	< 0.01*
Lean body mass (kg)	57.110.5	56.210.3	< 0.01*
Fat mass (kg)	13.33.1	13.42.9	0.66
Total body water (L)	42.18.5	41.38.1	< 0.01*

Presented are parameters of body composition in all participants that reached the 70- or 100-km checkpoint. *Significant differences pre- to post-race.

### Serum Analysis

Serum parameter of FIN and NON-are depicted in Supplementary Table 3.

#### Cardiac and Skeletal Muscle Cell Markers

**FIGURE 1:**
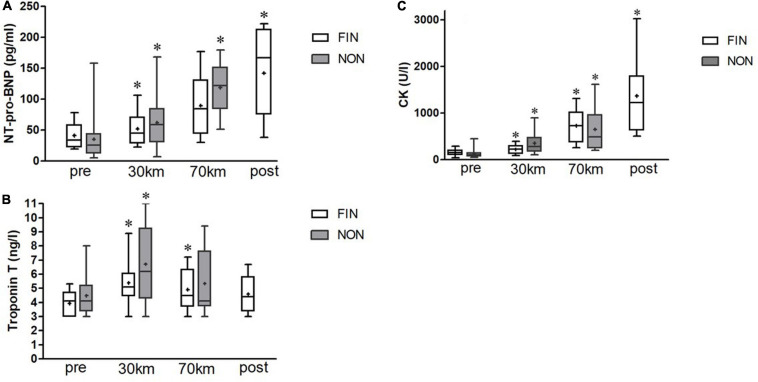
NT-pro-BNP **(A)**, troponin T **(B)**, and creatine kinase (CK) **(C)** alterations in finishers (FIN) and non-finishers (NON). Boxes represent 25th to 75th percentile, and whiskers minimum to maximum. Horizontal line shows median, and + shows mean. *Shows significant alterations to the previous checkpoint (paired *t*-tests, *p* < 0.05).

In both groups, serum levels of NT-pro-BNP, CK, and CK-MB increased significantly over the course of the MM. There were no significant differences for means at certain checkpoints between NON-and FIN (Figures 1A–C). Interestingly, mixed-effects ANOVA for troponin T showed significant results, driven by a significant increase only at the 30-km checkpoint (*p* < 0.05), but not at 70 and 100 km (Figure 1B).

#### Fat Metabolism

**FIGURE 2:**
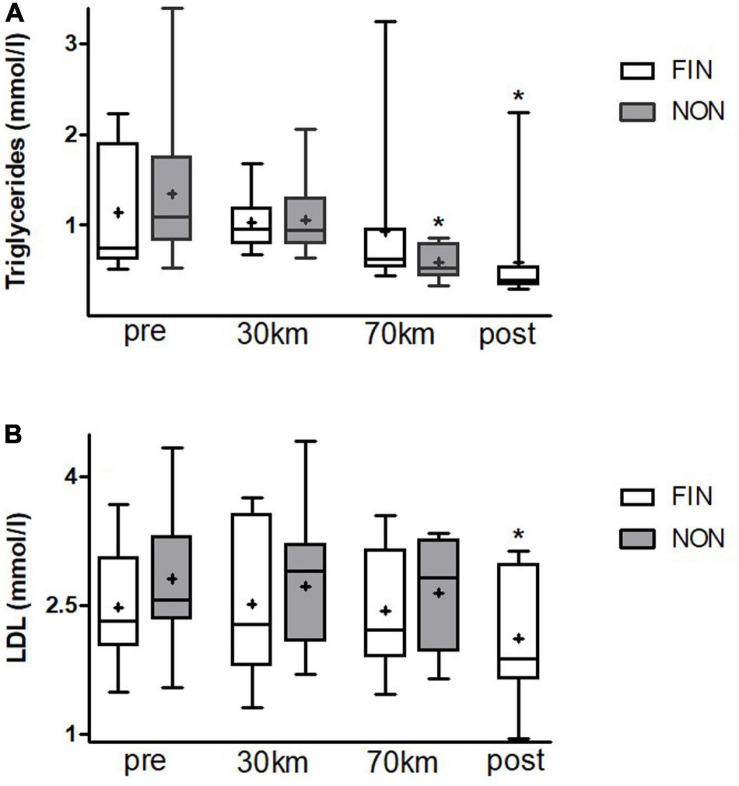
Triglyceride **(A)** and LDL **(B)** alterations in FIN and NON. Boxes represent 25th to 75th percentile, and whiskers minimum to maximum. Horizontal line shows median, and + shows mean. *Shows significant alterations to the previous checkpoint (paired *t*-tests, *p* < 0.05).

Triglycerides decreased significantly over the duration of the MM by over 50% (Figure 2A) in NON-and in FIN as a strong trend (*p* < 0.06). LDL decreased by approximately 20% as a strong trend in FIN (*p* = 0.05) (Figure 2B), while HDL increased significantly in NON (Supplementary Table 1). Paired *t*-tests showed a significant decrease after 70 km for cholesterol, LDL, and triglycerides as well as a significant HDL increment (*p* < 0.05 for all).

#### Stress and Inflammatory Markers

**FIGURE 3:**
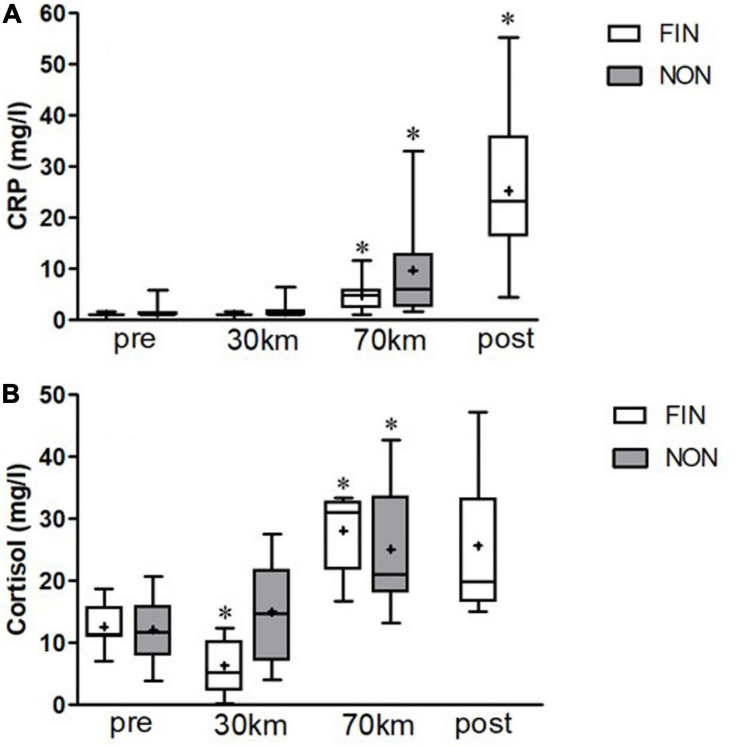
C-reactive protein (CRP) **(A)** and cortisol **(B)** alterations in FIN and NON. Boxes represent 25th to 75th percentile, and whiskers minimum to maximum. Horizontal line shows median, and + shows mean. *Shows significant alterations to the previous checkpoint (paired *t*-tests, *p* < 0.05).

C-reactive protein increased significantly over the course of the MM in both groups starting at 70 km (Figure 3A). Cortisol levels also changed significantly in both groups (Figure 3B). Of interest, at 30 km, the cortisol level in NON-was significantly higher than that in FIN, since the cortisol level initially declined in the FIN group.

#### Sodium Alterations

**FIGURE 4:**
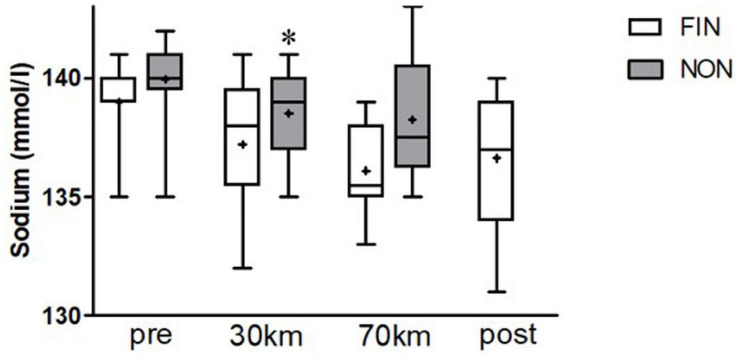
Sodium alterations in FIN and NON. Boxes represent 25th to 75th percentile, and whiskers minimum to maximum. Horizontal line shows median, and + shows mean. *Shows significant alterations to the previous checkpoint (paired *t*-tests, *p* < 0.05).

Sodium levels declined in both FIN and NON-groups, while a stronger decrease was observed in the FIN group up to 70 km distance (Figure 4).

### Correlation Analysis

#### Metabolic Equivalent of Task

Out of the 20 participants wearing actimeter armbands, 15 delivered usable serum samples (*n* = 15) for correlation analysis [7 out of 11 finishing participants (FIN) and 8 of 21 non-finishers (NON)]. From those participants, serum samples were gathered for correlation analysis. Mean MET did not differ significantly between groups or sex (Supplementary Table 2).

Data from the SenseWear armbands showed that participants had a higher MET in the first quarter of the race (18:00–24:00) in comparison to the last three quarters: mean MET from 18:00 to 24:00 h was 5.2, and from 24:00 to 18:00 h the next day, 4.4 (*p* < 0.01). Five MET was chosen as a cut-off value for a most even distribution. Participants who exhibited a higher mean MET (MET > 5) finished the track in a shorter time and walked with a higher speed as a trend [3.95 ± 0.89 vs. 4.36 ± 0.47 km/h (*p* = 0.32)]. Mean MET for subjects with a mean MET < 5 was 4.54 ± 0.31; for MET > 5, it was 5.25 ± 0.18.

**FIGURE 5:**
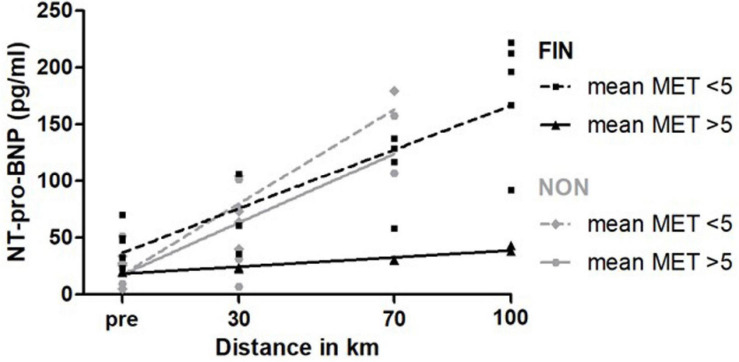
NT-pro-BNP over MET in FIN and NON. The black line represents the regression line in FIN, and the gray line represents the regression line in NON.

Participants with mean MET < 5 showed a significantly higher slope (= higher increase) for NT-pro-BNP (Figure 5) and CK-MB in FIN. In NON, CK increased significantly greater for participants with lower mean MET (Supplementary Table 3).

Our data indicate that NT-pro-BNP increased stronger in non-finishing participants (NON). The slope for NT-pro-BNP was significantly greater in NON-for participants that walked with a mean MET > 5 than in FIN (Table 3).

**TABLE 3:** NT-pro-BNP over MET subgroups dependent on outcome.

**Linear regression**		**MET < 5**		**MET > 5**	
		**FIN**	**NON**	**FIN**	**NON**
n		5	4	2	4
NT-pro-BNP	*r*^2^	0.67	0.85	0.93	0.64
(pg/ml)	Slope	1.30 ± 0.78	2.08 ± 0.33	0.21 ± 0.02	1.52 ± 0.40
	Elevation	36.86 ± 15.21	17.60 ± 10.06	18.19 ± 1.45	17.63 ± 14.80
	*p* (lines different?)	Slope: 0.17	Elevation: 0.98	Slope: < 0.01	Elevation: n.c.

*Presented are linear regression parameters for NT-pro-BNP in participants exercising with a mean MET > 5 and < 5 in FIN and NON. Presented are goodness of fit (*r*^2^), slope ± SD, elevation ± SD, and *p* values for slope and elevation to determine whether regression lines are significantly different. NT-pro-BNP levels rise significantly stronger in NON in the subgroup of MET > 5, shown by a significantly greater slope.*

#### Speed

Concerning speed, participants were divided into two subgroups: mean speed >4.3 km/h (mean: 4.65 ± 0.15 km/h) and <4.3 km/h (mean: 3.65 ± 0.72 km/h); 4.3 km/h was chosen as cut-off for a most even distribution. Mean speed in FIN (4.39 ± 0.26 km/h) was greater as a trend than in NON (3.87 ± 0.98 km/h; *p* = 0.19). There were no significant differences regarding sex, neither in FIN nor NON.

**FIGURE 6:**
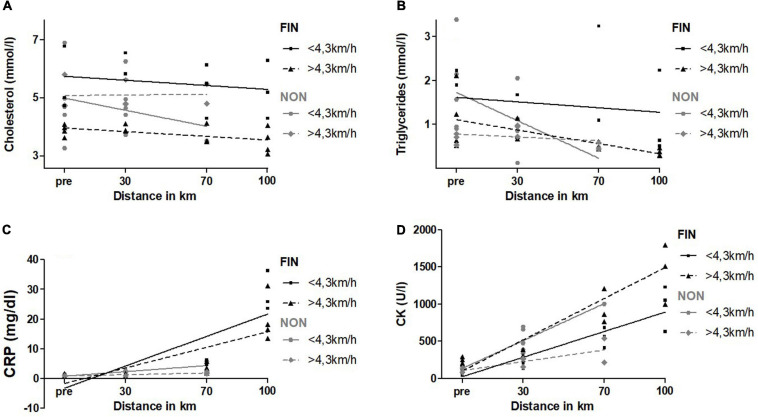
Cholesterol **(A)**, triglycerides **(B)**, CRP **(C)**, and CK **(D)** over speed in FIN and NON. The black line represents the regression line in FIN, and the gray line represents the regression line in NON.

Slower participants had significantly higher cholesterol and triglyceride levels in FIN (Figures 6A,B). CRP increased significantly greater in slower participants in NON (Figure 6C). CK levels were significantly greater in FIN for faster subjects, as opposed to NON, where slower participants expressed a greater increase of CK (Figure 6D). Exact values are presented in Supplementary Table 4.

In the subgroup of faster participants > 4.3 km/h, finishers showed a significantly greater increment of CK than non-finishers (slope FIN: 13.9 ± 2.0; slope NON: 3.9 ± 1.3; *p* < 0.01). In participants with a mean speed <4.3 km/h, NON-had significantly greater CK levels (elevation) with a similar slope to FIN (elevation FIN: 21.0 ± 89.2; elevation NON: 169.5 ± 66.1; *p* = 0.04).

For all the other parameters, there were no significant changes, or linear regression was not a fitting model.

#### Age

**FIGURE 7:**
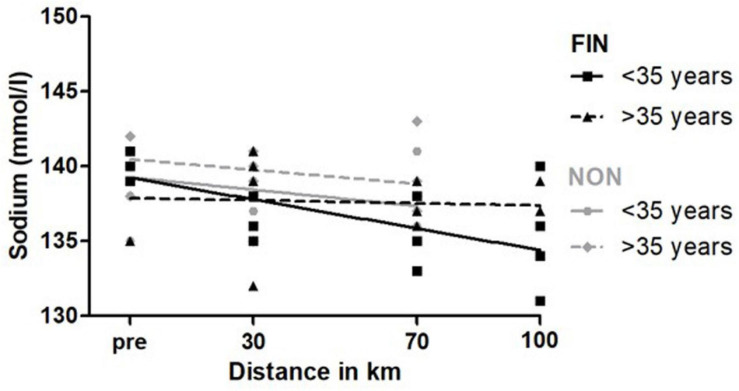
Sodium over speed in FIN and NON. The black line represents the regression line in FIN, and the gray line represents the regression line in NON.

Mean age in FIN was 35.7 ± 9.7 years; in NON, it was 34.3 ± 9.0 years, so 35 years was chosen as the cut-off for a most even distribution, in order to test for an age-related effect. Mean age for participants older than 35 years was 41.6 ± 6.2 years; for participants younger than 35, it was 27.9 ± 5.4. Linear regression analysis showed significant differences for an increase in CK and for a decrease in sodium (Supplementary Table 5). In FIN, older participants showed a significant greater increase for CK values. Sodium decreased significantly more in younger subjects in FIN. In NON, younger subjects had a significantly lower elevation in sodium (Figure 7).

For all the other parameters tested, there were no significant changes, or linear regression was not a fitting model.

#### BMI

**FIGURE 8:**
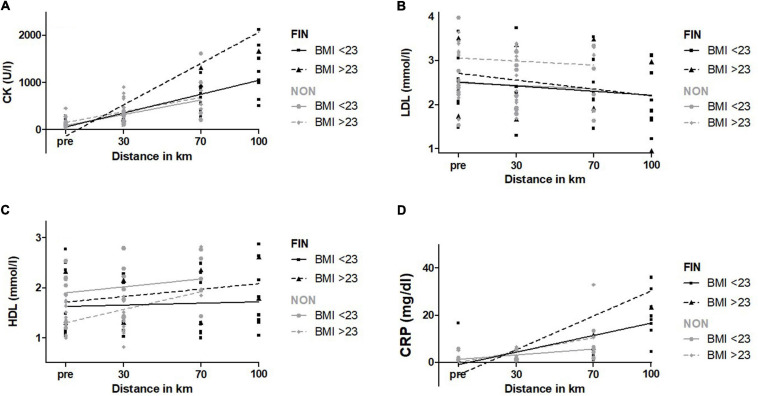
CK **(A)**, LDL **(B)**, HDL **(C)**, and CRP **(D)** over BMI in FIN and NON. The black line represents the regression line in FIN, and the gray line represents the regression line in NON.

Concerning BMI, 23 kg/m^2^ was chosen as cut-off for a most even distribution. Mean BMI in the group > 23 kg/m^2^ was 25.4 ± 2.0 kg/m^2^; in the group < 23 kg/m^2^, it was 21.4 ± 1.3 kg/m^2^. Linear regression analysis showed a significantly higher increase for CK, CK-MB, and AST for participants with a higher BMI in FIN (*p* < 0.01) (Figure 8A). In NON, participants with higher BMI showed significantly higher elevations of LDL and lower HDL levels. Slopes did not differ significantly (Figures 8B,C). CRP increased stronger in participants with a higher BMI as a trend in both FIN and NON (*p* < 0.10) (Figure 8D). Exact values are presented in Supplementary Table 6.

#### Sex

**FIGURE 9:**
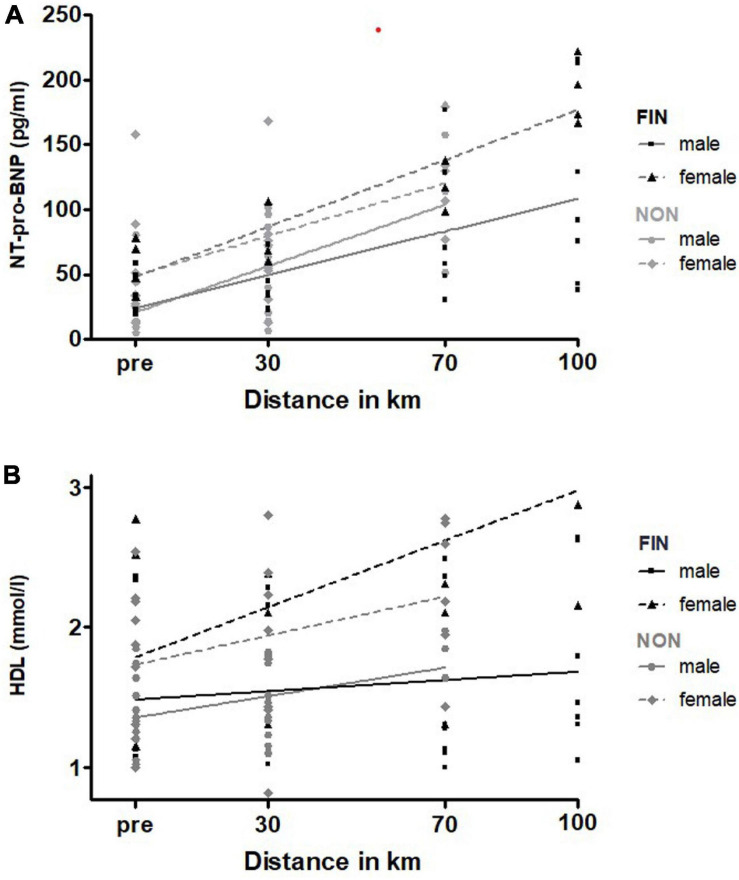
NT-pro-BNP **(A)** and HDL **(B)** over sex in FIN and NON. The black line represents the regression line in FIN, and the gray line represents the regression line in NON.

All serum parameters were analyzed with linear regression analysis for significant differences in male and female. NT-pro-BNP and HDL showed significantly higher elevations in females of both FIN and NON-groups (Figures 9A,B). Baseline NT-pro-BNP was significantly higher in females (54.20 ± 10.60 pg/ml) as confirmed by *t*-test (male: 25.9 ± 4.6 pg/ml; *p* = 0.01). Supplementary Table 7 depicts the exact results.

For all the other parameters, there were no significant changes, or linear regression was not a fitting model.

#### Baseline NT-pro-BNP

**FIGURE 10:**
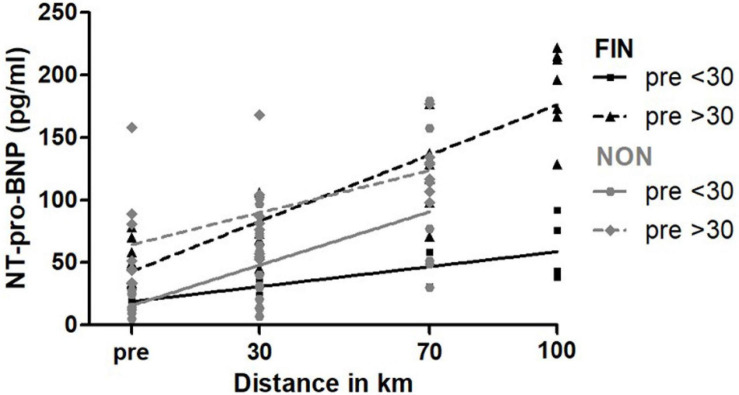
NT-pro-BNP over baseline NT-pro-BNP in FIN and NON. The black line represents the regression line in FIN, and the gray line represents the regression line in NON.

It was also evaluated whether a higher baseline NT-pro-BNP > 30 pg/ml led to different changes in serum parameters than a lower baseline NT-pro-BNP < 30 pg/ml with linear regression analysis. NT-pro-BNP values for participants with higher baseline NT-pro-BNP increased relatively stronger, shown by a significantly higher slope in linear regression analysis in FIN (Supplementary Table 8 and Figure 10).

In the subgroup of participants with a baseline NT-pro-BNP < 30 pg/ml, subjects who were not able to finish the race (NON) had a significantly greater increment of NT-pro-BNP over the course of the MM, compared with FIN (slope NON: 1.37 ± 0.24; slope FIN: 0.30 ± 0.09; *p* < 0.01). In subjects with a baseline NT-pro-BNP > 30 pg/ml, there were no significant changes. Finally, heart rate did not differ significantly for groups with regard to NT-pro-BNP.

#### Mean Heart Rate During the Race

All serum parameters were analyzed with linear regression analysis for significant differences between participants that had a mean heart rate <106.5 and >106.5 bpm during the race; 106.5 bpm was the median and chosen as the cut-off value. Mean heart rate during the race in FIN was 106.8 ± 8.5 bpm; in NON, it was 108.5 ± 13.4 bpm (*p* = 0.79).

**FIGURE 11:**
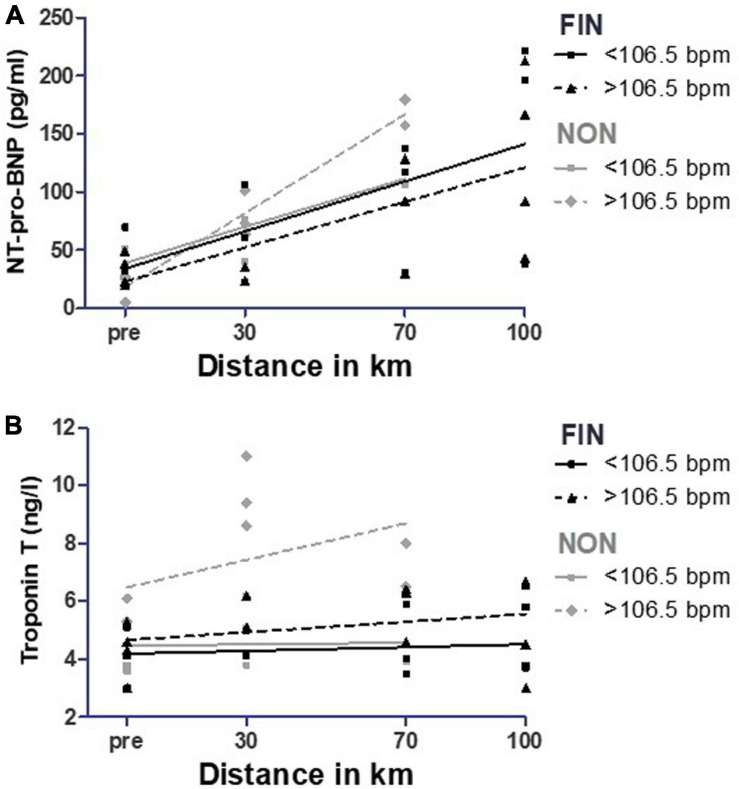
NT-pro-BNP **(A)** and troponin T **(B)** over mean heart rate during the race in FIN and NON. The black line represents the regression line in FIN, and the gray line represents the regression line in NON.

**FIGURE 12:**
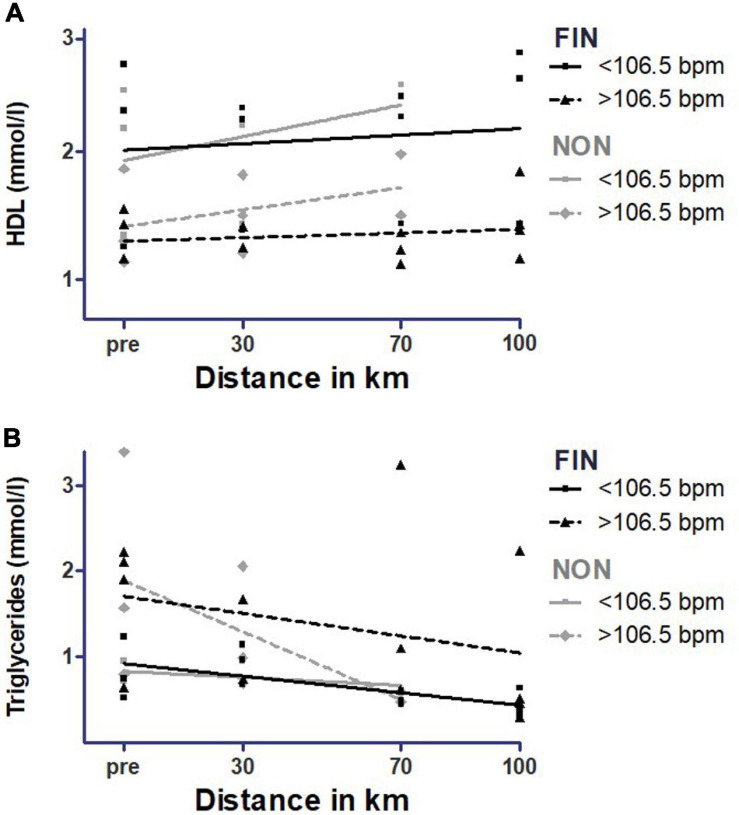
High-density lipoprotein **(A)** and triglycerides **(B)** over mean heart rate during the race in FIN and NON. The black line represents the regression line in FIN, and the gray line represents the regression line in NON.

In NON, NT-pro-BNP showed a significantly higher increase in participants with a mean heart rate > 106.5 during the MM (*p* = 0.02) (Figure 11A and Supplementary Table 9). Participants with a greater heart rate during the MM > 106.5 bpm showed significantly higher levels for troponin T (*p* = 0.02) and higher levels for triglycerides as a trend (*p* = 0.11) (Figures 11B, 12B and Supplementary Table 9). In FIN, participants with greater heart rate during the race had significantly higher values of triglycerides (*p* = 0.02), and troponin T levels were greater as a trend (*p* < 0.10) (Figures 11B, 12B and Supplementary Table 9). In both FIN and NON, subjects with lower heart rates during the race had significantly higher elevations of HDL (*p* < 0.01 in FIN and *p* = 0.02 in NON) (Figure 12A and Supplementary Table 9).

## Discussion

This study was conducted to evaluate whether a low-intensity long-endurance march, during which participants were walking instead of running or jogging, would elicit similar changes in body composition, metabolism, and cardiovascular risk markers as described previously in higher intensity marathons or ultramarathons (Ginsburg et al., 1996; Fallon, 2001; Wu et al., 2004; Scharhag et al., 2005, 2006; Knechtle et al., 2008a; Scott et al., 2009; Kasprowicz et al., 2013; Kłapcińska et al., 2013; Marklund et al., 2013; Kim et al., 2014; Salvagno et al., 2014; Vilela et al., 2015; Baur et al., 2016; Emed et al., 2016; Yoon et al., 2016; Coker et al., 2017). To our knowledge, this is the first investigation to evaluate a broad range of physiological parameters among healthy human adults in such a unique setting. Data from actimeter armbands showed that the MM was conducted at a lower mean speed (4.12 km/h) and therefore lower intensity (mean MET ≈5) than in classical (ultra-) marathons, conducted with jogging and running speeds of 8–14 km/h (Scharhag et al., 2005; La Gerche et al., 2008) and a mean energy expenditure of 8–10 (Mendes et al., 2018); in addition, the average in-race heart rate (≈106–108 bpm) was comparatively low.

The considerable amount of distances covered on foot per week (≈35–55 km), a good self-evaluated endurance capacity (≈7.3 out of 10) combined with the low-normal mean resting heart rates (≈67 bpm) (Kannel et al., 1987), and a normal mean BMI (≈22–25 kg/m^2^; Global Bmi Mortality Collaboration et al., 2016) suggest that our study participants were aerobically fit and healthy recreational athletes, but not highly trained endurance athletes who display much lower resting heart rates of below 50 bpm (Doyen et al., 2019) and, in severe cases, even below 30 bpm (Jensen-Urstad et al., 1997).

It is a noteworthy finding that despite a greater amount of distance covered on foot per week by the female subjects of our study in both FIN and NON, the self-rated endurance capability was lower as a trend among women compared with men in FIN and differed significantly between men and women in NON (8.0 ± 0.8 vs. 6.4 ± 1.3; *p* = 0.02). This supports previous findings that women underestimate their capabilities in general (Beyer, 1990) and their physical performance in particular (Obling et al., 2015). Further research appears warranted with regard to self-perceived gender differences among marathon and ultramarathon runners.

We hypothesized that the low working intensity of the evaluated “Mammutmarsch,” during which participants were required to walk instead of running or jogging, would cause no elevation of muscle and cardiac stress markers among our study participants. However, measurements of several physiological parameters as well as testing for the various serum stress markers indicated that, opposite to our working hypothesis, significant changes in stress parameters, such as CRP, CK, CK-MB, AST, ALT, cortisol, and NT-pro-BNP, have occurred, as well as in parameters of lipid metabolism (cholesterol, LDL, HDL, and triglycerides). As further discussed below, we conclude that the distance, intensity, and most importantly duration determine the exertion and, thus, the physiological impact.

### Body Composition and Hydration Status

As previously shown by other studies (Knechtle et al., 2008a; Baur et al., 2016), we observed a significant decrease in total body mass (−1%, *p* < 0.01) over the course of this low-intensity long-endurance event. In contrast to the higher intensity multistage ultramarathons described by Baur et al. (2016) and Knechtle et al. (2008a), the weight loss in our study was caused by a loss of fat-free mass (−1.6%, *p* < 0.01) and a decrease in total body water (TBW; −1.9%, *p* < 0.01), respectively. In contrast, the mentioned studies found a significant loss in fat mass [24.4% (Baur et al., 2016) and 42.7% (Knechtle et al., 2008a)] with no significant changes in TBW (Baur et al., 2016) and lean body mass or even a significant increase in TBW (Knechtle et al., 2008a). Knechtle et al. (2008b) described a correlation between intensity and loss of body fat during the Triple Iron Triathlon Germany 2006 in Lensahn. Participants with a better performance during the MM showed a moderately larger decrease in fat mass. This effect is also described in laboratory settings; in their study with 18 moderately trained cyclists taking part in continuous prolonged exercise tests, Achten et al. (2002) found that with higher intensity, there is an increase in fat oxidation, up to a maximum fat oxidation rate at an intensity of 64 ± 4% of VO_2_max. Nevertheless, Coker et al. (2017) described that during the Yukon Arctic Ultramarathon, which also takes place at lower intensities, in this case with an average speed of 2–3 mph (corresponding to 3.2–4.8 km/h), fat loss occurred. It should be mentioned that in this setting participants marched up to 430 miles (≈690 km) in 7–14 days in adverse climate conditions with temperatures reaching down to −45°C. Thus, participants suffered from exercise-induced caloric expenditure for a much longer period of time, with chronic exposure to extreme cold as an additional burden. Also in the studies of Baur et al. (2016) and Knechtle et al. (2008a), participants exercised 30 to more than 48 h in two to three consecutive days. Alpert (2005) described 290 kJ/kg per day the maximal transfer rate of energy from FM. With a mean FM of approximately 13.3 kg in this study, this results in a maximal possible fat loss of roughly 120 g, assuming 7,700 kcal (about 32,000 kJ) is needed to burn 1 kg of body fat (Wishnofsky, 1958). Thus, most likely the maximum duration of 24 h was not enough to exhibit a significant loss of FM, regardless of the intensity. But even under these extreme settings, several studies reported high levels of muscle preservation (Knechtle et al., 2008a; Saugy et al., 2013; Coker et al., 2017).

From the fact that the loss of body mass in our study is mainly a loss of TBW, associated with muscle preservation in even more extreme settings (Knechtle et al., 2008a; Saugy et al., 2013; Coker et al., 2017), we conclude that a loss of body mass is most likely a consequence of sweating in connection with insufficient rehydration. This assumption is supported by our data showing a decrease in serum sodium levels over the course of the MM that resulted in a mild hyponatremia in six participants (minimum: 131 mmol/L). This could be interpreted as a consequence of dehydration and rehydration with large amounts of fluids, resulting in dilution hyponatremia (Knechtle and Nikolaidis, 2018).

Nevertheless, sodium levels reached no critical values, implying adequate rehydration. Supportingly, Noakes et al. (2005) described a loss of body mass < 3% as a status of euhydration.

Linear regression analysis showed that younger participants had a stronger decrease of sodium. A possible explanation for this might be training status. In their review, Knechtle and Nikolaidis (Knechtle and Nikolaidis, 2018) suggested an age of 30–50 to be the “best” years to take part in an ultramarathon. The longer the distance, the better the achievements of the older participants were (Knechtle et al., 2014). This implies adaptation to long-term training (Knechtle and Nikolaidis, 2018). Additionally, it is scientific consensus that aging reduces the sensitivity of sweat glands to acetylcholine, leading to a reduced sudomotor function (Lee et al., 2016). Therefore, by sweating more, younger participants might have lost more sodium than older participants. Furthermore, it is widely accepted that older subjects feel less thirst and therefore drink later (Arai et al., 2014). Thus, we speculate that there was less sodium loss and blood dilution, explaining the relatively higher sodium levels in older participants.

### NT-pro-BNP

Synthesis of NT-pro-BNP is triggered mechanically by ventricle stretching and neurohumoral stimulation (Nakagawa et al., 1995). Among other cardiac stress markers, NT-pro-BNP increased significantly in every single participant. Over the course of the march, there was almost a fourfold increment from 37.4 to 142.2 pg/ml. Notably, NT-pro-BNP values of > 125 pg/ml can indicate acute congestive heart failure (Maisel et al., 2002; Cowie et al., 2003).

Elevation of NT-pro-BNP after and during long-endurance events as a cardiac stress parameter is well described in several studies (Scharhag et al., 2005; Scott et al., 2009; Banfi et al., 2012; Kłapcińska et al., 2013; Kim et al., 2014; Salvagno et al., 2014). In a 48-h run with a mean distance of 170 km, NT-pro-BNP increased 10-fold in the first 24 h from 54 to 508 pg/ml (Kłapcińska et al., 2013). In male athletes, Scharhag et al. described a threefold increase (40–130 pg/ml) after a marathon, a sixfold increase (40–250 pg/ml) in a 100-km ultramarathon, and a fourfold increase (20–80 pg/ml) in a 110-km mountain bike marathon (Scharhag et al., 2005). These events were running or biking events; therefore, intensity levels were higher, compared with our study. In a longer low-intensity 308-km run, Yoon et al. (2016) reported an almost 10-fold increase (27–257 pg/ml).

It is one of the main findings of our study that also a low-intensity long-endurance march like the MM leads to changes in NT-pro-BNP levels comparable to higher intensity long-endurance running events. Currently, the majority of studies (Scharhag et al., 2005, 2008; Banfi et al., 2012; Kłapcińska et al., 2013) interpret increasing NT-pro-BNP levels as part of a process of myocardial adaption rather than a harmful effect (La Gerche et al., 2008). Moreover, recent studies described that the duration of the exercise might have a higher influence on NT-pro-BNP levels than exercise intensity (Legaz-Arrese et al., 2011; Serrano-Ostáriz et al., 2011). Comparing our study to longer low-intensity endurance events, showing a greater NT-pro-BNP increment, our data supports this hypothesis (Kłapcińska et al., 2013; Yoon et al., 2016). A possible explanation for this phenomenon was given by Legaz-Arrese et al.: NT-pro-BNP release is triggered by ventricle stretching and therefore preload changes. Higher intensities result in higher heart rates and shorter R-R intervals, so preload stretch is limited and intensity and preload do not increase in a linear fashion (Nakagawa et al., 1995; Legaz-Arrese et al., 2011). However, the aforementioned studies were conducted at much higher intensities at above 85% of their participant’s anaerobic threshold (Legaz-Arrese et al., 2011; Serrano-Ostáriz et al., 2011).

Interestingly, participants who could only sustain a lower mean MET showed a greater increment of NT-pro-BNP. Furthermore, in the subgroup of participants exercising with a mean MET > 5, non-finishing participants showed significantly stronger increasing NT-pro-BNP values. This raises the question whether NT-pro-BNP might be a marker for cardiovascular capacity also in healthy adults. Our data indicate that NT-pro-BNP values increased stronger in participants with higher heart rates during the race (>106.5 bpm) in NON, underpinning this theory, as heart rate is inversely correlated with cardiorespiratory fitness (Kang et al., 2017). Moreover, recent studies showed that higher NT-pro-BNP levels were associated with lower cardiorespiratory fitness in patients with chronic heart disease (Nichols et al., 2018). Scharhag et al. (2006) found a similar result with a negative correlation between the increase in NT-pro-BNP and the amount of endurance training per week in healthy athletes: participants with higher training volumes showed lower increases in NT-pro-BNP, one given explanation was a better adapted myocardium on endurance exercise bouts. A greater increment for NT-pro-BNP in participants with higher baseline NT-pro-BNP values observed in finishing participants of this study supports this hypothesis. All in all, our data suggest that even at low intensities as observed in our study, NT-pro-BNP increases take place among healthy adults that might indicate a cardiac stimulation and may thus represent possible cardiac training previously seen only at higher intensities (Vilela et al., 2015). Nevertheless, to gain unambiguous data, further studies are needed, as, e.g., the subgroup of participants with a lower mean MET did not show significant differences between FIN and NON.

### Troponin T

Exercise-induced troponin elevations after prolonged physical activity have been described in several studies (Scharhag et al., 2005, 2006, 2008; Scott et al., 2009; Eijsvogels et al., 2010; Legaz-Arrese et al., 2011; Serrano-Ostáriz et al., 2011; Banfi et al., 2012; Kłapcińska et al., 2013; Kim et al., 2014; Salvagno et al., 2014; Arakawa et al., 2016; Yoon et al., 2016; Knechtle and Nikolaidis, 2018). The clinical significance is still debated. While some authors postulate exercise-induced irreversible myocardial injury, cell necrosis, or damage (Koller et al., 1999; Eijsvogels et al., 2010), others presume a reversible release of cytoplasmic troponins due to membrane leakage or changes in intracellular calcium levels without any pathological consequence (Scharhag et al., 2005, 2008; Shave et al., 2007; Benda et al., 2016). Interestingly, some studies on ultramarathons indicated no increase of troponin T (Kim et al., 2014). In this study, we observed a significant elevation of troponin T after the 30-km distance but found normal levels at the 70- and 100-km checkpoints. A similar troponin dynamic was found in a 48-h ultramarathon (Kłapcińska et al., 2013). This might show an adaption process to the prolonged myocardial and volume stress. Another possible explanation might be the impact of intensity. The activity data suggests that participants started with a significantly higher mean MET in the first third of the race possibly leading to troponin T elevation. Similar observations were made in the 48-h ultramarathon, in that subjects ran with a higher mean speed in the first 12 h (8.6 km/h) compared with the next 12 h (6.0 km/h) (Kłapcińska et al., 2013). With exhaustion-induced lowering of walking or running speed in the last two thirds of the race, intensity might be below the exertion level causing troponin T excretion.

This conclusion is supported by Scharhag et al. (2005). They compared a marathon, mountain bike marathon, and a 100-km ultramarathon. Median troponin T concentrations increased significantly during the marathon and the mountain bike marathon only, but not during the lower intensity 100-km ultramarathon. Supporting this theory, Shave et al. (2007) suggested a cardiac troponin correlation to intensity, as shown in their meta-analysis, where athletes in shorter long-endurance events, such as marathons, expressed the highest troponin elevations.

Our study supports the hypothesis of an intensity-related release of cardiac troponins. Furthermore, greater troponin T elevations in subjects with higher heart rate during the race support the findings of Mehta et al. of an inverse correlation to training status (Mehta et al., 2012). Further studies are warranted to gain more understanding into the release of cardiac troponins and exercise intensity and duration.

### Markers for Cell Damage

Aminotransferase, CK, and CK-MB are muscle cell enzymes, released by the breakdown of skeletal muscle (Khan, 2009). For all, levels increased significantly over the course of the MM. CK reached levels more than 10 times above the URL, which indicates severe muscular damage. CK-MB and AST increased fourfold and threefold, respectively. A generally accepted explanation is cell leakage due to mechanical tissue damage (Kłapcińska et al., 2013).

Increases of muscle stress markers after long-endurance exercise were described in several studies (Scharhag et al., 2005; Banfi et al., 2012; Waśkiewicz et al., 2012; Kłapcińska et al., 2013; Kim et al., 2014; Arakawa et al., 2016; Baur et al., 2016; Yoon et al., 2016; Knechtle and Nikolaidis, 2018). Training status, sex, duration, BMI, age, and intensity are considered as an impact factor for interindividual CK elevation (Neal et al., 2009; Banfi et al., 2012; Waśkiewicz et al., 2012; Kłapcińska et al., 2013; Yoon et al., 2016; Kim and So, 2018; Nichols et al., 2018).

In our study, CK and CK-MB levels increased continuously over distance, reaching its maximum level at 100 km or the individual point of dropping out of the MM. Studies covering ultramarathons observed a strong variance in CK levels: Scharhag et al. (2005) and Yoon et al. (2016) described relatively moderate CK elevations after a marathon and 100 km ultramarathon, although participants ran with a higher running speed (around 10 km/h). In contrast, in a 308-km ultramarathon, CK levels reached 5,270.06 U/L, an almost 35-fold increment (Yoon et al., 2016). Waśkiewicz et al. (2012) described CK elevations up to 17,500 U/L in a 24-h ultramarathon with a mean speed of 7 km/h. Such large CK elevations were also observed by Kłapcińska et al. (2013) after running 24 h with a mean speed of 7.3 km/h, reaching 18,000 U/L. It is worth mentioning that in the studies of Kłapcińska et al. (2013) and Waśkiewicz et al. (2012), amateur runners were tested, while Scharhag et al. (2005) and Yoon et al. (2016) assessed experienced endurance athletes. In our study, recreational athletes were recruited and CK increased to a greater degree in all participants with a mean MET < 5. Strikingly, this contradicts the view of intensity being the relevant impact factor. Nevertheless, it can be argued that athletes who were only able to walk with a lower mean MET are possibly less trained and therefore training status has the major impact (Banfi et al., 2012).

We observed that age, BMI, and sex impact CK elevation in the low-intensity MM. Older participants with a higher BMI and males compared with females showed higher CK elevations. Males had a higher BMI in this study, so there might be a confounding effect. Nevertheless, higher BMI is described as an impact factor for CK elevation (Neal et al., 2009; Banfi et al., 2012; Kim and So, 2018). For age as an impact factor, variable results exist. As assessed in a review (Baird et al., 2012), some authors describe age as a factor contributing to greater exercise-induced damage, and others report lower CK elevations, pointing to age-related sarcopenia as a cause.

Aminotransferase is generally accepted as a marker for skeletal muscle cell damage after long-endurance exercise (Banfi et al., 2012; Waśkiewicz et al., 2012; Kłapcińska et al., 2013). On average, AST increased approximately 2.5-fold, while ALT increased only marginally. In this study, increases of AST are interpreted as a marker for skeletal muscle cell damage and not liver cell damage, as also suggested by other studies (Banfi et al., 2012; Waśkiewicz et al., 2012; Kłapcińska et al., 2013). In our cohort, AST levels were further associated with higher age, as well as higher BMI, which supports data previously reported in the literature (Banfi et al., 2012).

Like CK, CK-MB is also known as a marker for muscle cell damage, in skeletal muscles as well as in the myocardium (Kim et al., 2014; Yoon et al., 2016; Knechtle and Nikolaidis, 2018). As it is also a marker for myocardial cell damage, it is to be discussed whether CK-MB increment in this setting is also representative for cardiac injury. Over the course of the race, mean CK-MB rose approximately 4.5-fold, while CK levels increased almost 10-fold. Studies suggested that the considerably CK increases represented predominantly a skeletal muscle damage also because in the abovementioned studies no cardiac injury was reported (Kim et al., 2014; Knechtle and Nikolaidis, 2018). Normal troponin T levels at 70 and 100 km support the hypothesis of skeletal muscle damage instead of cardiac cell damage or even necrosis.

### Stress Parameters

C-reactive protein increased significantly by the end of the MM and reached values 25 times higher than the baseline. After 70 km, CRP increased from 1–2 to 7 mg/L, reaching 25 mg/L after 100 km. CRP elevation as acute phase response marker after long-endurance exercise has been described by multiple other studies (Fallon, 2001; Waśkiewicz et al., 2012; Kasprowicz et al., 2013; Kłapcińska et al., 2013; Baur et al., 2016). IL-6 is widely described as the inducing element of CRP production in the liver, which plays an anti-inflammatory role (induction of anti-inflammatory cytokines) and targets and induces removal of damaged cells (Waśkiewicz et al., 2012; Kłapcińska et al., 2013). Duration and intensity are the main factors in CRP elevation (Waśkiewicz et al., 2012; Kłapcińska et al., 2013). In a 3-day multistage ultra-endurance triathlon in which subjects exercised for a total of roughly 30 h with resting periods in between the stages, CRP increased from <1 to 9 mg/L (Baur et al., 2016). Untrained subjects in the study of Kłapcińska et al. (2013) showed an increment from <1 to 30 mg/L after running for 24 h with a mean speed of roughly 7.3 km/h. Fallon (2001) observed similar dynamics in a track race: CRP increased from 1.9 to 21.9 mg/L after 24 h, covering a distance of 142.6 km/h. The greatest increment was found in a 24-h ultramarathon in which subjects ran with a mean speed of 7 km/h: CRP pre-race was 1.7 mg/L, 8.7 mg/L after 12 h, and reached 39.2 mg/L after 24 h (Waśkiewicz et al., 2012). All subjects in the cited studies ran with a higher running speed than those in this study. Nevertheless, CRP increased in a comparable fashion in this low-intensity march.

Cortisol showed a significant dynamic over the course of the march. Changing cortisol levels can be interpreted as part of its physiological circadian rhythm (Edwards et al., 2001) as serum samples have been taken over the course of 24 h. Nevertheless, values after 70 km distance of the MM reached levels that are outside the URL, showing a neuroendocrine answer to this long-endurance low-intensity event. Studies describe cortisol elevations as a response to stress through physical exercise, inducing a neuroendocrine answer, which is necessary to conserve energy for vital functions (by suppression of the hypothalamo–pituitary–gonadal axis) and to increase substrate availability (cortisol-induced gluconeogenesis) (Popovic et al., 2019).

### Lipid Profile

Triglyceride serum concentrations decreased significantly over the course of the MM in FIN and NON. Cholesterol and LDL decreased significantly in FIN. Notably, the mixed-effects ANOVA showed only nearly significant results, as cholesterol and LDL levels decreased to a large extent only after 70 km. Triglyceride levels (baseline: 1.27–1.33 mmol/L) started to decrease already after 30 km (1.05 mmol/L) and reached 0.59 mmol/L after 100 km. Paired *t*-tests showed that LDL and cholesterol significantly decreased from 70 to 100 km, dropping by roughly 20% and approximately 12%, respectively. Additionally, HDL levels (baseline: 1.61 mmol/L) increased significantly at 70 km (2.05 mmol/L) in NON, and the increment in FIN started only after 70 km rising to 1.86 mmol/L post-march. Favorable shifts in blood lipid levels during ultramarathons are well established in the literature (Ginsburg et al., 1996; Wu et al., 2004; Banfi et al., 2012; Waśkiewicz et al., 2012; Emed et al., 2016; Knechtle and Nikolaidis, 2018). We found that females showed higher elevations of HDL, corroborating the findings in the current literature (Davis et al., 1996).

In the higher intensity Hawaii Ironman Triathlon in which athletes exercised for a total of approximately 12.5 h, Ginsburg et al. (1996) observed a 9% decrease of cholesterol, a 39% decrease of triglyceride levels, and a 10% decrease of LDL. HDL did not change significantly. In a 24-h ultramarathon, in which participants ran with a mean speed of 7 km/h for almost 170 km, cholesterol decreased from 5.07 to 4.04 mmol/L, LDL from 2.86 to 1.74 mmol/L, and triglycerides from 1.73 to 0.80 mmol/L, and HDL increased from 1.43 to 2.01 mmol/L (Waśkiewicz et al., 2012).

Notably, the lipid profile changes in our study were comparable to triathlons or ultramarathons of much higher intensities.

Long-endurance events like ultramarathons cause a high caloric demand, and carbohydrates as a source of energy have limited stores in the body (Coyle et al., 1986). Hence, an increase of fat oxidation, resulting in the mobilization of storage fat, is needed to cover the energy expenditure (Waśkiewicz et al., 2012; Kłapcińska et al., 2013). Since blood lipid profile with high cholesterol levels is established as a main risk factor for chronic heart disease, it can be argued that long-endurance exercise should be considered beneficial for the prevention of cardiovascular diseases (Knechtle and Nikolaidis, 2018). Aadahl et al. (2007) described a linear correlation between moderate physical activity and cardiovascular risk factors. Mann et al. (2014) also described a linear dose–response relationship between activity levels and HDL cholesterol levels. To reduce LDL and triglyceride levels, higher intensities have been reported to be required (Mann et al., 2014). Especially aerobic exercise at high intensities is reported to be effective in improving the LDL/HDL ratio (Mann et al., 2014). Fikenzer et al. (2018) even mentioned a minimum exercise threshold to produce beneficial effects on serum lipids. In contrast, however, a decrement in cholesterol and LDL levels was also observed after 70 km in this long-endurance low-intensity setting, suggesting that a constant low-intensity activity such as during the MM might be as effective as an influence on lipid profiles as shorter high-intensity exercises that have been previously reported.

Nevertheless, even in this 100-km march of low-intensity, participants who walked with greater speed showed more favorable lipid profiles than slower participants. A possible explanation could be a better training status in faster individuals, resulting in a higher mean walking speed, as endurance training is considered beneficial for lipid status (Knechtle and Nikolaidis, 2018), but also due to a higher rate of fat oxidation found among better trained endurance athletes (Bassett and Howley, 2000). Also in this study, FIN had a greater history of endurance training as distance covered on foot per week as a trend. In addition, the less favorable triglyceride levels among subjects with greater heart rate and the more favorable HDL levels in participants with lower heart rate during the race support this explanation. The fact that participants of FIN walked with a greater walking speed as a trend (4.39 vs. 3.79 km/h, *p* = 0.19) adds to this as well.

This study also showed the expected correlation between greater BMI and higher plasma lipid levels (Waśkiewicz et al., 2012). Eijsvogels and Maessen (2017) described striking risk reductions for all-cause and cardiovascular mortality already at low volumes of physical activity, with additional health benefits with increasing exercise volumes. Especially for subjects with a higher BMI or cardiac disabilities who are not able to train with higher intensities, low-intensity but longer endurance exercise might lead to benefits. In conclusion, our study coincides with clinical trials, suggesting health benefits concerning the lipid profile from low-intensity long-endurance exercise.

### General Remarks and Limitations

Even though this study showed highly significant results, the low number of participants limits the validity, especially in FIN and in correlation analyses of subgroups. In some cases, FIN and NON-did not show the same results or did not reach the same level of significance. This might be due to the low number of participants and lower statistical power of FIN. Nevertheless, the overall number of 43 participants (32 with usable serum samples) in that kind of experimental setting in a field study conducted over 3 years in our investigation is relatively high, as most studies assessing ultramarathons covered 5–35 participants (Fallon, 2001; Wu et al., 2004; Scharhag et al., 2005; Knechtle et al., 2008a,b; La Gerche et al., 2008; Scott et al., 2009; Waśkiewicz et al., 2012; Kasprowicz et al., 2013; Kłapcińska et al., 2013; Marklund et al., 2013; Saugy et al., 2013; Kim et al., 2014; Salvagno et al., 2014; Baur et al., 2016; Emed et al., 2016; Yoon et al., 2016; Coker et al., 2017). The differentiation into FIN and NON, even though numbers in those subgroups were small, allowed us to perform a differential analysis of performance to detect possible influencing factors.

A different training status among subjects needs to be considered as a potential influencing factor as well. Even though subjects of FIN did not exhibit a statistically significant greater amount of distance covered per week, our assessment points in that direction as a trend.

Another influencing factor might be each participant’s nutrition or hydration plan, which was not assessed. This could have had an influence on blood parameters. To minimize the influence, participants were instructed to neither eat nor drink at least 1 h before each checkpoint.

Both speed and MET were used for correlation analysis, as no linear correlation was to be expected because of the individuals’ heterogenic conditions. A well-trained participant with low BMI can express a lower mean MET and still walk faster than an untrained, heavier person and vice versa. The different results in these analyses confirm this assumption.

Furthermore, we would like to point out that data was acquired over 3 years in three different MM events. Nevertheless, weather and GPS data show that these races took place under similar conditions, and consequently, we consider the acquired data as comparable.

Serum samples were stored for up to 24 h in a sealed cooling box before further processing. The analyzed parameters should not be affected by this; nevertheless, some serum samples were not usable due to hemolysis, thereby reducing the statistical power.

### Summary

Despite the low walking speed and hence low intensity, at which the MM was conducted, our data reveal significant changes among healthy human adults in body composition, metabolism, and stress parameters that were comparable to those observed in ultramarathons of much higher intensity and different kind of athletes. Participants lost around 1% of body mass, mostly from a significant loss of body water (-1.9%), while the duration of 24 h was not long enough to reduce fat mass significantly.

NT-pro-BNP showed a significant elevation over time and distance, indicating that even at such low intensities a sufficient cardiac stimulation might have taken place. The duration of low-intensity activity turned out to be the main impact factor. Despite reaching values above the URL, no evidence for cardiac cell damage was found, as troponin T values increased only moderately in the first third of the race, later returning to baseline values. Other than for NT-pro-BNP, intensity seemed to have been the predominant factor for exercise-induced troponin T release. As performance and heart rate during the race correlated with the increment of NT-pro-BNP values, NT-pro-BNP might be a marker for cardiovascular fitness also in healthy individuals. Training status seems to have correlated with cardiac stress markers as well. Further studies are needed to confirm this hypothesis.

In addition, this low-intensity march elicited massive changes in markers for cell damage, showing skeletal muscle damage leading to an acute inflammatory response. Current literature suggests a quick cell repair and return to baseline values after exercise.

The MM triggered shifts to a favorable lipid profile comparable to endurance events of much higher intensity. Also, for cholesterol, duration seems to have the greatest influence, as LDL and cholesterol started to decrease significantly only after 70 km. It is a clinically relevant finding that a low-intensity exercise can have an impact on serum lipids, as subjects with pre-existing conditions might not be able to exercise at higher intensities.

In addition, even though none of our participants were professional endurance athletes, our results suggest that a more active lifestyle, as the trend-wise greater distance covered on foot per week among FIN, will lead to a more favorable lipid profile and better performance.

In conclusion, long-endurance marches like the 100-km MM can lead to striking increases in stress metabolism markers. Thus, such long-duration events could not be recommended for the general population to improve cardiovascular fitness. Nevertheless, in the light of increasing prevalence of metabolic diseases associated with a sedentary lifestyle (Arocha Rodulfo, 2019), which can be construed to be a negative environmental factor on the health of a large proportion of the population in western societies (Lin et al., 2020), the results of our study might help to bring about health benefits by changing the lifestyle from a sedentary to a more active one (e.g., walking instead of driving, using the stairs instead of the elevator, etc.). Since our data suggest that even exercise at low intensities such as walking can have the reported positive benefits, our study supports several recommendations already incorporated in general guidelines (Anton et al., 2011) and could further help clinicians apply lifestyle changes for the health benefits of their patients.

## Data Availability Statement

The raw data supporting the conclusions of this article will be made available by the authors, without undue reservation.

## Ethics Statement

The study was approved by the Charité Ethics Board (review number EA1/163/14). Written informed consent to participate in this study was provided by the participants’ legal guardian/next of kin.

## Author Contributions

MJ contributed in writing the manuscript, performed the measurements, and obtained and analyzed the data. MS designed, planned and implemented the study, performed the measurements, and obtained the data. HG and MS provided the critical expertise, feedback, and revised the final manuscript. All authors contributed to the article and approved the submitted version.

## Conflict of Interest

The authors declare that the research was conducted in the absence of any commercial or financial relationships that could be construed as a potential conflict of interest.
