# Supplementary material for: Physiological Changes, Activity, and Stress During a 100-km–24-h Walking-March
Source: Front Physiol. 2021 Mar 11;12:640710. doi: 10.3389/fphys.2021.640710 (PMC7991843; doi:10.3389/fphys.2021.640710)
Supplement: Supplementary file 1 [file Data_Sheet_1.docx]

**Supplementary Tables**

**Supplementary Table 1: Serum parameters in FIN and NON groups.**

**Supplementary Table 1: Serum parameters in FIN and NON groups.** *Provided are means ± SD. Unpaired t-tests serve to indicate significance between FIN and NON per checkpoint. Mixed-effects ANOVA (anova) indicates significant differences over the course of the race per parameter in each group.*

| Serum: FIN/NON | **pre** | | |  | **30km** |  | **70km** | **100km** | **p** |
| --- | --- | --- | --- | --- | --- | --- | --- | --- | --- |
|  |  |  | p (t-test) |  | p (t-test) |  | p (t-test) |  | (anova) |
| NT-pro-BNP (pg/ml) | FIN | 41.4 ± 20.8 | 0.61 | 52.16 ± 27.6 | 0.49 | 89.8 ± 49.7 | 0.16 | 142.2 ± 70.0 | <0.01 |
|  | NON | 35.3 ± 36.2 |  | 62.1 ± 38.3 |  | 118.9 ± 41.5 |  | - | <0.01 |
| Troponin T (ng/l) | FIN | 3.9 ± 0.9 | 0.25 | 5.4 ± 1.6 | 0.17 | 4.9 ± 1.4 | 0.64 | 4.6 ± 1.4 | <0.01 |
|  | NON | 4.5 ± 1.4 |  | 6.7 ± 2.6 |  | 5.3 ± 2.3 |  | - | 0.02 |
| CK (U/I) | FIN | 156.1 ± 76.1 | 0.26 | 222.9 ± 96.6 | 0.23 | 729.2 ± 365.2 | 0.31 | 1368 ± 766.5 | <0.01 |
|  | NON | 130.1 ± 89.6 |  | 354.0 ± 228.7 |  | 651.1 ± 483.4 |  | - | <0.01 |
| CK-MB (U/I) | FIN | 11.5 ± 3.1 | 0.17 | 16.9 ± 2.7 | 0.88 | 28.2 ± 14.5 | 0.78 | 47.2 ± 41.8 | <0.03 |
|  | NON | 9.7 ± 3.5 |  | 16.6 ± 5.8 |  | 30.1 ± 13.8 |  | - | <0.01 |
| CRP (mg/l) | FIN | 1.1 ± 0.2 | 0.24 | 1.1 ± 0.2 | 0.21 | 4.8 ± 3.0 | 0.17 | 25.36 ± 13.8 | <0.01 |
|  | NON | 1.5 ± 1.3 |  | 1.7 ± 1.5 |  | 9.8 ± 10.3 |  | - | <0.01 |
| Cortisol (µg/dl) | FIN | 12.6 ± 3.3 | 0.78 | 6.4 ± 4.3 | <0.01 | 28.1 ± 6.0 | 0.44 | 25.7 ± 11.1 | <0.01 |
|  | NON | 12.1 ± 5.1 |  | 15.0 ± 7.3 |  | 25.1 ± 9.9 |  | - | <0.01 |
| Cholesterol (mg/dl) | FIN | 180.5 ± 40.1 | 0.45 | 181.3 ± 43.9 | 0.74 | 174.5 ± 40.5 | 0.32 | 164.2 ± 42.7 | 0.1 |
|  | NON | 191.2 ± 36.5 |  | 186.1 ± 31.5 |  | 193.1 ± 34.9 |  | - | 0.13 |
| LDL (mg/dl) | FIN | 96.6 ± 27.0 | 0.22 | 98.1 ± 34.9 | 0.5 | 94.9 ± 27.4 | 0.52 | 82.5 ± 29.7 | 0.05 |
|  | NON | 109.3 ± 27.7 |  | 106.1 ± 26.6 |  | 103.1 ± 25.5 |  | - | 0.18 |
| HDL (mg/dl) | FIN | 65.6 ± 25.9 | 0.54 | 65.2 ± 63.9 | 0.77 | 63.9 ± 23.3 | 0.13 | 72.5 ± 24.4 | 0.1 |
|  | NON | 60.9 ± 16.8 |  | 63.0 ± 18.0 |  | 79.9 ± 17.6 |  | - | <0.01 |
| Triglycerides (mg/dl) | FIN | 101.1 ± 57.0 | 0.43 | 91.2 ± 26.4 | 0.82 | 82.7 ± 52.0 | 0.28 | 52.0 ± 49.3 | 0.06 |
|  | NON | 118.9 ± 61.4 |  | 93.9 ± 30.5 |  | 52.63 ± 17.8 |  | - | <0.01 |
| AST (U/I) | FIN | 23.6 ± 4.3 | 0.68 | 27.8 ± 3.7 | 0.83 | 37.8 ± 12.3 | 0.81 | 56.6 ± 26.2 | <0.01 |
|  | NON | 22.8 ± 5.7 |  | 28.4 ± 8.1 |  | 36.4 ± 12.9 |  | - | <0.01 |
| ALT (U/I) | FIN | 21.5 ± 4.6 | 0.7 | 22.0 ± 5.4 | 0.68 | 21.4 ± 6.3 | <0.05 | 25.6 ± 6.7 | <0.03 |
|  | NON | 20.2 ± 10.1 |  | 20.4 ± 10.6 |  | 15.4 ± 4.3 |  | - | 0.85 |
| Sodium (mmol/l) | FIN | 139.0 ± 2.1 | 0.16 | 137.2 ± 2.8 | 0.12 | 136.1 ± 1.9 | 0.06 | 136.6 ± 3.0 | 0.05 |
|  | NON | 140.0 ± 1.6 |  | 138.5 ± 1.7 |  | 138.3 ± 2.7 |  | - | 0.02 |

**Supplementary Table 2: Mean MET in FIN and NON.**

**Supplementary Table 2: Mean MET in FIN and NON.** *Displayed are mean MET of female and male subjects in FIN and NON. There were no significant differences.*

| **mean MET** | | | | |
| --- | --- | --- | --- | --- |
|  | n | male | female | p |
| FIN | 7 | 4.85 | 4.83 | 0.96 |
| NON | 13 | 4.98 | 4.56 | 0.14 |
| p |  | 0.64 | 0.46 |  |

**Supplementary Table 3: Cardiac and skeletal muscle markers dependent on MET in FIN and NON.**

**Supplementary Table 3: Cardiac and skeletal muscle markers dependent on MET in FIN and NON.** *Presented are linear regression parameters for NT-pro-BNP, CK and CK-MB in FIN and NON dependent on exercise intensity (MET >5 and <5). Presented are goodness of fit (r²), slope ± SD, elevation ± SD and p-values for slope and elevation to determine whether regression lines are significantly different.*

| linear regression |  | **FIN** | | **NON** | |
| --- | --- | --- | --- | --- | --- |
|  |  | MET <5 | MET >5 | MET <5 | MET >5 |
| n |  | 5 | 2 | 4 | 4 |
| NT-pro-BNP | r² | 0.69 | 0.93 | 0.85 | 0.64 |
| (pg/ml) | slope | 1.30 ± 0.22 | 0.21 ± 0.02 | 2.08 ± 0.33 | 1.52 ± 0.40 |
|  | elevation | 36.86 ± 14.64 | 18.19 ± 01.45 | 17.60 ± 10.06 | 17.63 ± 14.80 |
|  | p (lines different?) | slope: <0.01 | elevation: n.c. | slope: 0.32 | elevation: 0.34 |
| CK | r² | 0.69 | 0.90 | 0.82 | 0.28 |
| (U/L) | slope | 12.79 ± 2.20 | 8.49 ± 1.16 | 11.44 ± 2.03 | 4.19 ± 2.35 |
|  | elevation | 58.07 ± 143.5 | 113.6 ± 72.66 | 121.1 ± 62.43 | 172.8 ± 86.01 |
|  | p (lines different?) | slope: 0.23 | elevation: 0.25 | slope: <0.05 | elevation: n.c. |
| CK-MB | r² | 0.88 | 0.90 | 0.88 | 0.53 |
| (U/L) | slope | 0.28 ± 0.03 | 0.15 ± 0.02 | 0.35 ± 0.05 | 0.26 ± 0.09 |
|  | elevation | 9.73 ± 1.77 | 10.75 ± 1.27 | 7.43 ± 1.49 | 9.05 ± 3.13 |
|  | p (lines different?) | slope: <0.01 | elevation: n.c. | slope: 0.40 | elevation: 0.84 |

**Supplementary Table 4: Serum parameters dependent on mean speed in FIN and NON.**

**Supplementary Table 4: Serum parameters dependent on mean speed in FIN and NON.** *Presented are linear regression parameters for cholesterol, triglycerides, CK and CRP in FIN and NON dependent on speed (<4.3 km/h and >4.3 km/h). Presented are goodness of fit (r²), slope ± SD, elevation ± SD and p-values for slope and elevation to determine whether regression lines are significantly different.*

| linear regression |  | **FIN** | | **NON** | |
| --- | --- | --- | --- | --- | --- |
| mean speed |  | <4.3 km/h | >4.3 km/h | <4.3 km/h | >4.3 km/h |
| n |  | 3 | 4 | 5 | 3 |
| Cholesterol | r² | 0.04 | 0.25 | 0.08 | 0.01 |
| (mg/dl) | slope | -0.18 ± 0.27 | -0.16 ± 0.08 | -0.54 ± 0.61 | -0.04 ± 0.21 |
|  | elevation | 223.6 ± 17.6 | 154.0 ± 5.1 | 194.2 ± 17.9 | 198.4 ± 9.3 |
|  | p (lines different?) | slope: 0.95 | elevation: <0.01 | slope: 0.44 | elevation: 0.30 |
| Triglycerides | r² | 0.02 | 0.43 | 0.26 | 0.11 |
| (mg/dl) | slope | -0.68 ± 0.23 | -0.30 ± 0.63 | -1.89 ± 1.05 | -0.19 ± 0.23 |
|  | elevation | 142.7 ± 40.7 | 97.6 ± 14.6 | 152.8 ± 30.8 | 68.8 ± 8.9 |
|  | p (lines different?) | slope: 0.53 | elevation: <0.02 | slope: 0.15 | elevation: 0.15 |
| CRP | r² | 0.66 | 0.60 | 0.59 | 0.69 |
| (mg/dl) | slope | 0.25 ± 0.06 | 0.17 ± 0.04 | 0.05 ± 0.01 | 0.01 ± 0.00 |
|  | elevation | -3.2 ± 3.9 | -1.6 ± 2.6 | 0.8 ± 0.2 | 0.8 ± 0.4 |
|  | p (lines different?) | slope: 0.30 | elevation: 0.42 | slope: <0.01 | elevation: n.c. |
| CK | r² | 0.80 | 0.80 | 0.86 | 0.60 |
| (U/l) | slope | 8.7 ± 1.4 | 13.9 ± 2.0 | 12.5 ± 1.7 | 3.9 ± 1.3 |
|  | elevation | 25.7 ± 93.23 | 103.9 ± 129.0 | 137.4 ± 49.4 | 107.0 ± 52.2 |
|  | p (lines different?) | slope: 0.06 | elevation: <0.01 | slope: <0.01 | elevation: n.c. |

**Supplementary Table 5: Serum parameters dependent on age in FIN and NON.**

**Supplementary Table 5: Serum parameters dependent on age in FIN and NON.** *Presented are linear regression parameters for CK and Sodium in FIN and NON dependent on age (<35 years and >35 years). Presented are goodness of fit (r²), slope ± SD, elevation ± SD and p-values for slope and elevation to determine whether regression lines are significantly different.*

| linear regression |  | **FIN** | | **NON** | |
| --- | --- | --- | --- | --- | --- |
| age |  | <35 years | >35 years | <35 years | >35 years |
| n |  | 5 | 6 | 13 | 8 |
| CK | r² | 0.56 | 0.63 | 0.25 | 0.57 |
| (U/l) | slope | 7.7 ± 1.8 | 15.8 ± 2.6 | 6.5 ± 2.12 | 8.4 ± 1.7 |
|  | elevation | 83.92 ± 114.2 | -41.50 ± 160.4 | 129.3 ± 68.7 | 138.1 ± 62.9 |
|  | p (lines different?) | slope: 0.02 | elevation: n.c. | slope: 0.50 | elevation: 0.43 |
| Sodium | r² | 0.46 | 0.01 | 0.12 | 0.11 |
| (mmol/l) | slope | -0.05 ± 0.01 | 0.0 ± 0.01 | -0.03 ± 0.01 | -0.02 ± 0.02 |
|  | elevation | 139.2 ± 0.9 | 137.9 ± 0.82 | 139.3 ± 0.43 | 140.5 ± 0.6 |
|  | p (lines different?) | slope: 0.03 | elevation: n.c. | slope: 0.85 | elevation: 0.01 |

**Supplementary Table 6: Serum parameters dependent on BMI in FIN and NON.**

**Supplementary Table 6: Serum parameters dependent on BMI in FIN and NON.** *Presented are linear regression parameters for CK, CK-MB, AST, CRP, LDL and HDL in FIN and NON dependent on BMI (<23 kg/m² and >23 kg/m²). Presented are goodness of fit (r²), slope ± SD, elevation ± SD and p-values for slope and elevation to determine whether regression lines are significantly different.*

| linear regression |  | **FIN** | | **NON** | |
| --- | --- | --- | --- | --- | --- |
| BMI |  | <23 kg/m² | >23 kg/m² | <23 kg/m² | >23 kg/m² |
| n |  | 9 | 2 | 7 | 14 |
| CK | r² | 0.56 | 0.78 | 0.33 | 0.44 |
| (U/l) | slope | 9.9 ± 1.6 | 22.0 ± 4.8 | 7.8 ± 2.7 | 7.6 ± 1.6 |
|  | elevation | 54.1 ± 100.8 | -137.1 ± 303.8 | 83.7 ± 107.6 | 150.4 ± 47.2 |
|  | p (lines different?) | slope: <0.01 | elevation: n.c. | slope: 0.95 | elevation: 0.39 |
| CK-MB | r² | 0.79 | 0.32 | 0.44 | 0.58 |
| (U/l) | slope | 0.23 ± 0.02 | 0.89 ± 0.40 | 0.28 ± 0.08 | 0.29 ± 0.05 |
|  | elevation | 10.0 ± 1.5 | 2.0 ± 24.9 | 9.2 ± 3.0 | 9.0 ± 1.4 |
|  | p (lines different?) | slope: <0.01 | elevation: n.c. | slope: 0.83 | elevation: 0.91 |
| AST | r² | 0.61 | 0.56 | 0.18 | 0.40 |
| (U/l) | slope | 0.25 ± 0.04 | 0.65 ± 0.23 | 0.18 ± 0.08 | 0.22 ± 0.06 |
|  | elevation | 21.2 ± 2.3 | 18.0 ± 14.7 | 22.2 ± 3.1 | 22.8 ± 1.9 |
|  | p (lines different?) | slope: <0.01 | elevation: n.c. | slope: 0.70 | elevation: 0.52 |
| CRP | r² | 0.43 | 0.59 | 0.30 | 0.30 |
| (mg/l) | slope | 0.18 ± 0.04 | 0.36 ± 0.12 | 0.06 ± 0.02 | 0.15 ± 0.04 |
|  | elevation | -0.97 ± 2.33 | -5.32 ± 7.64 | 1.22 ± 0.94 | -0-08 ± 1.28 |
|  | p (lines different?) | slope: 0.06 | elevation: 0.21 | slope: 0.10 | elevation: 0.52 |
| LDL | r² | 0.03 | 0.04 | 0.01 | 0.00 |
| (mg/dl) | slope | -0.12 ± 0.12 | 0.20 ± 0.38 | -0.08 ± 0.21 | -0.09 ± 0.25 |
|  | elevation | 97.9 ± 7.7 | 105.5 ± 24.0 | 97.2 ± 8.5 | 119.1 ± 7.5 |
|  | p (lines different?) | slope: 0.79 | elevation: 0.76 | slope: 0.97 | elevation: 0.01 |
| HDL | r² | 0.00 | 0.08 | 0.05 | 0.25 |
| (mg/dl) | slope | 0.04 ± 0.11 | 0.14 ± 0.20 | 0.15 ± 0.16 | 0.34 ± 0.11 |
|  | elevation | 63.4 ± 6.8 | 66.8 ± 12.8 | 74.1 ± 6.5 | 50.8 ± 3.3 |
|  | p (lines different?) | slope: 0.67 | elevation: 0.35 | slope: 0.31 | elevation: <0.01 |

**Supplementary Table 7: Serum parameters dependent on sex in FIN and NON.**

**Supplementary Table 7: Serum parameters dependent on sex in FIN and NON.** *Presented are linear regression parameters for NT-pro-BNP and HDL in FIN and NON dependent on sex. Presented are goodness of fit (r²), slope ± SD, elevation ± SD and p-values for slope and elevation to determine whether regression lines are significantly different.*

| linear regression |  | **FIN** | | **NON** | |
| --- | --- | --- | --- | --- | --- |
|  |  | female | male | female | male |
| n |  | 4 | 7 | 9 | 12 |
| NT-pro-BNP | r² | 0.83 | 0.34 | 0.3 | 0.46 |
| (pg/ml) | slope | 1.29 ± 0.17 | 0.84 ± 0.24 | 1.02 ± 0.34 | 1.19 ± 0.26 |
|  | elevation | 48.1 ± 10.8 | 24.3 ± 15.1 | 49.0 ± 12.9 | 20.8 ± 7.9 |
|  | p (lines different?) | slope: 0.21 | elevation: <0.01 | slope: 0.70 | elevation: 0.02 |
| HDL | r² | 0.07 | 0.11 | 0.07 | 0.19 |
| (mg/dl) | slope | -0.01 ± 0.19 | 0.08 ± 0.11 | 0.21 ± 0.17 | 0.20 ± 0.08 |
|  | elevation | 79.5 ± 11.5 | 57.8 ± 7.2 | 68.1 ± 6.3 | 52.8 ± 2.5 |
|  | p (lines different?) | slope: 0.68 | elevation: 0.03 | slope: 0.93 | elevation: <0.01 |

**Supplementary Table 8: serum parameters dependent on baseline NT-pro-BNP in FIN and NON.**

**Supplementary Table 8: serum parameters dependent on baseline NT-pro-BNP in FIN and NON.** *Presented are linear regression parameters for NT-pro-BNP in FIN and NON dependent on sex. Presented are goodness of fit (r²), slope ± SD, elevation ± SD and p-values for slope and elevation to determine whether regression lines are significantly different.*

| linear regression |  | **FIN** | | **NON** | |
| --- | --- | --- | --- | --- | --- |
| baseline NT-pro-BNP | | <30 pg/ml | >30 pg/ml | <30 pg/ml | >30 pg/ml |
| n |  | 4 | 7 | 13 | 8 |
| NT-pro-BNP | r² | 0.56 | 0.77 | 0.3 | 0.46 |
| (pg/ml) | slope | 0.40 ± 0.10 | 1.34 ± 0.15 | 1.37 ± 0.24 | 0.77 ± 0.31 |
|  | elevation | 18.7 ± 6.0 | 42.9 ± 9.8 | 12.6 ± 7.6 | 65.2 ± 11.4 |
|  | p (lines different?) | slope: <0.01 | elevation: n.c | slope: 0.12 | elevation: <0.01 |

**Supplementary Table 9: serum parameters dependent on mean heart rate during the reace in FIN and NON.**

**Supplementary Table 9: serum parameters dependent on mean heart rate during the race in FIN and NON.** *Presented are linear regression parameters for NT-pro-BNP in FIN and NON dependent on sex. Presented are goodness of fit (r²), slope ± SD, elevation ± SD and p-values for slope and elevation to determine whether regression lines are significantly different.*

| linear regression |  | **FIN** | | **NON** | |
| --- | --- | --- | --- | --- | --- |
| mean heart rate (race) | | <106.5 bpm | >106.5 bpm | <106.5 bpm | >106.5 bpm |
| n |  | 3 | 4 | 3 | 3 |
| NT-pro-BNP | r² | 0.38 | 0.46 | 0.63 | 0.95 |
| (pg/ml) | slope | 1.07 ± 0.43 | 0.98 ± 0.32 | 1.03 ± 0.36 | 2.13 ± 0.20 |
|  | elevation | 34.15 ± 27.07 | 22.99 ± 21.05 | 39.13 ± 11.74 | 18.01 ± 7.74 |
|  | p (lines different?) | slope: 0.86 | elevation: 0.45 | slope: 0.02 | elevation: n.c. |
| Troponin T | r² | 0.02 | 0.09 | 0.00 | 0.16 |
| (ng/l) | slope | 0.00 ± 0.01 | 0.01 ± 0.01 | 0.00 ± 0.02 | 0.03 ± 0.03 |
|  | elevation | 4.19 ± 0.45 | 4.66 ± 0.56 | 4.47 ± 0.56 | 6.48 ± 1.15 |
|  | p (lines different?) | slope: 0.62 | elevation: <0.10 | slope: 0.44 | elevation: 0.02 |
| HDL | r² | 0.01 | 0.04 | 0.11 | 0.16 |
| (mg/dl) | slope | 0.07 ± 0.19 | 0.04 ± 0.06 | 0.27 ± 0.35 | 0.19 ± 0.18 |
|  | elevation | 78.46 ± 12.14 | 47.07 ± 4.04 | 74.90 ± 11.60 | 52.13 ± 7.17 |
|  | p (lines different?) | slope: 0.86 | elevation: <0.01 | slope: 0.83 | elevation: 0.02 |
| Triglycerides | r² | 0.42 | 0.09 | 0.06 | 0.33 |
| (mg/dl) | slope | -0.43 ± 0.16 | -0.58 ± 0.55 | -0.21 ± 0.37 | -1.76 ± 1.01 |
|  | elevation | 81.30 ± 9.90 | 150.8 ± 36.16 | 73.49 ± 12.16 | 167.1 ± 39.97 |
|  | p (lines different?) | slope: 0.80 | elevation: 0.02 | slope: 0.23 | elevation: 0.11 |
